# Impact of non-cardiovascular comorbidities on the quality of life of patients with chronic heart failure: a scoping review

**DOI:** 10.1186/s12955-020-01566-y

**Published:** 2020-10-07

**Authors:** Josep Comín-Colet, Teresa Martín Lorenzo, Almudena González-Domínguez, Juan Oliva, Silvia Jiménez Merino

**Affiliations:** 1 Community Heart Failure Program, Department of Cardiology, Hospital Universitario de Bellvitge; Bellvitge Biomedical Research Institute (IDIBELL), L’Hospitalet de Llobregat, Barcelona, Spain; 2 University of Barcelona, Barcelona, Spain; 3 Weber, Madrid, Spain; 4 Department of Economic Analysis, University of Castilla-La Mancha, Toledo, Spain; 5 Vifor Pharma, Barcelona, Spain

**Keywords:** Heart failure, Health-related quality of life, Comorbidity, Chronic disease

## Abstract

**Purpose:**

To determine the impact of non-cardiovascular comorbidities on the health-related quality of life (HRQoL) of patients with chronic heart failure (CHF).

**Methods:**

A scoping review of the scientific literature published between 2009 and 2019 was carried out. Observational studies which assessed the HRQoL of patients with CHF using validated questionnaires and its association with non-cardiovascular comorbidities were included.

**Results:**

The search identified 1904 studies, of which 21 fulfilled the inclusion criteria to be included for analysis. HRQoL was measured through specific, generic, or both types of questionnaires in 72.2%, 16.7%, and 11.1% of the studies, respectively. The most common comorbidities studied were diabetes mellitus (12 studies), mental and behavioral disorders (8 studies), anemia and/or iron deficiency (7 studies), and respiratory diseases (6 studies). Across studies, 93 possible associations between non-cardiovascular comorbidities and HRQoL were tested, of which 21.5% regarded anemia or iron deficiency, 20.4% mental and behavioral disorders, 20.4% diabetes mellitus, and 14.0% respiratory diseases. Despite the large heterogeneity across studies, all 21 showed that the presence of a non-cardiovascular comorbidity had a negative impact on the HRQoL of patients with CHF. A statistically significant impact on worse HRQoL was found in 84.2% of associations between mental and behavioral disorders and HRQoL (patients with depression had up to 200% worse HRQoL than patients without depression); 73.7% of associations between diabetes mellitus and HRQoL (patients with diabetes mellitus had up to 21.8% worse HRQoL than patients without diabetes mellitus); 75% of associations between anemia and/or iron deficiency and HRQoL (patients with anemia and/or iron deficiency had up to 25.6% worse HRQoL than between patients without anemia and/or iron deficiency); and 61.5% of associations between respiratory diseases and HRQoL (patients with a respiratory disease had up to 21.3% worse HRQoL than patients without a respiratory disease).

**Conclusion:**

The comprehensive management of patients with CHF should include the management of comorbidities which have been associated with a worse HRQoL, with special emphasis on anemia and iron deficiency, mental and behavioral disorders, diabetes mellitus, and respiratory diseases. An adequate control of these comorbidities may have a positive impact on the HRQoL of patients.

## Introduction

Chronic heart failure (CHF) is a disease caused by structural or functional cardiac abnormalities that result in reduced cardiac output and/or increased cardiac pressure at rest or stress [1, 2]. In developed countries, the prevalence of CHF is estimated at 1–3% of the adult population, exceeding 10% in people over 70, and 30% in people over 85 [3]. Due to the aging of the population, the prevalence of CHF is estimated to increase by 46% in 2030 compared to 2012 in the United States [4].

Patients with CHF often have multiple comorbidities, both cardiovascular and non-cardiovascular, which accelerate disease progression, to a greater or lesser extent, and worsen the response to treatment [5, 6]. However, while most deaths are due to cardiovascular causes, non-cardiovascular causes (chronic renal failure, anemia, diabetes) are responsible for most hospitalizations [5, 7–10]. Likewise, it is known that patients with non-cardiovascular comorbidities present a higher risk of mortality and increased length of hospitalization compared to patients with CHF without comorbidities or those with only cardiovascular comorbidities [5, 7, 8, 11, 12].

Different studies have observed that the non-cardiovascular comorbidities with the highest prevalence among patients with CHF include iron deficiency (prevalence of 53–65% [13]), anemia (prevalence of up to 37% [14]), diabetes mellitus (prevalence of between 23% [15] and 47% [16]), renal failure (prevalence of up to 55% [14]), depression (prevalence of up to 61% [17]), and respiratory diseases (prevalence of up to 63% [18]), among others. The presence of these comorbidities and their association with higher rates of hospitalization and length of hospitalization could lead to a significant deterioration in the functional capacity and health-related quality of life (HRQoL) of patients with CHF.

Recently, a systematic literature review identified multiple factors associated with the HRQoL of patients with heart failure (HF), including the presence of comorbidities [19]. However, the relationship between non-cardiovascular comorbidities and HRQoL was not clearly established, as it was not the main objective of the study. Other studies have aimed at documenting or analyzing the impact of one or more non-cardiovascular comorbidities on the HRQoL of patients with CHF compared to healthy subjects [8, 20–22]. However, there are methodological differences in these studies, in terms of instruments used to measure HRQoL, statistical methods used to estimate measures of impact, and differences in the choice of comorbidities of interest. These differences complicate classifying the published evidence and obtaining accurate conclusions. A scoping review to outline the available evidence on the impact of non-cardiovascular comorbidities on the HRQoL in patients with CHF would provide the basis for future research to guide clinical practice on this matter. To our knowledge, this is the first paper to summarize previous studies focused on the impact of non-cardiovascular comorbidities on the HRQoL in patients with CHF.

The main objective of this scoping review was to identify and describe the available evidence on the impact that non-cardiovascular comorbidities have on the HRQoL of patients with CHF. Accordingly, our research question was: In patients with CHF, does the presence of non-cardiovascular comorbidities significantly worsen their HRQoL?

## Methodology

This scoping review has been developed according to the Preferred Reporting Items for Systematic Review and Meta-Analysis Extension for Scoping Reviews methodology [23]. Study selection, data extraction, and study quality ratings were performed by two independent reviewers. Moreover, a third reviewer was used to resolve any discrepancies between the first two.

### Search strategy

A systematic search of the scientific literature published in the electronic databases PubMed™/MEDLINE™ and Embase™ between January 1, 2009 and December 31, 2018 was carried out in January 2019. In addition, the Cochrane Database of Systematic Reviews was used to complete the search with articles included in relevant systematic literature reviews or meta-analysis. We restricted the search to studies published after 2009 in order to include only those that analyzed the impact of comorbidities on HRQoL in the context of the current management of patients with CHF.

The search was performed using a combination of search terms from Medical Subject Headings, Emtree™, and free text terms associated with "observational studies", "systematic reviews", "multicenter studies", "heart failure", and "quality of life". These terms, together with synonyms and abbreviations, were combined using the AND and OR operators. Specific terms for HRQoL ("quality of life", "health-related quality of life", "QALY", "quality-adjusted life years", "health utilities", "utility") were used, as well as questionnaires validated for overall HRQoL assessment (European Quality of Life-5 Dimensions [EQ-5D], 36-item Short Form Health Survey [SF-36], 12-item Short Form Health Survey [SF-12] and Short Form Six Dimension [SF-6D]) or specific for HF (Kansas City Cardiomyopathy Questionnaire [KCCQ] and Minnesota Living with Heart Failure Questionnaire [MLHFQ]). The search was limited to human studies published in English. Additional file 1 shows the search performed on each database.

### Inclusion and exclusion criteria and study selection

The articles obtained in each search were imported into the EndNoteX8 reference manager software (Clarivate Analytics, Philadelphia, PA, USA). Subsequently, duplicate articles were identified and removed from the list of results. We revised articles by title, abstract, and/or full text, and selected those that fulfilled the following inclusion criteria: complete original articles published in a scientific journal, published on or after 2009, published with the full text in English, studies carried out in countries with a long tradition in the field of health technology assessment and the development of HRQoL measurement instruments (Canada, United States, United Kingdom, European Union, Australia, and New Zealand); observational studies, multicenter studies, systematic reviews, studies related to HF as a primary diagnosis, studies that evaluate the HRQoL of patients with HF through validated questionnaires, and studies that test the relationship between the presence of a non-cardiovascular comorbidity and HRQoL.

In order to allow minimal comparability between studies, we decided to exclude studies related to acute HF, studies evaluating the HRQoL of caregivers of patients with HF, studies whose focus was outside the purpose of this scoping review (those that did not use questionnaires to assess the association between at least one non-cardiovascular comorbidity and the HRQoL of patients with HF), short reports of any research, conference abstracts, and any type of grey literature. We considered non-cardiovascular comorbidities of HF those included in the current guide of the European Society of Cardiology for the diagnosis and treatment of acute HF and CHF that were not considered cardiovascular comorbidities in the International Classification of Diseases and Related Health Problems 10th Revision [24].

### Data extraction

We designed a template in Microsoft® Excel® (Version 1905, Microsoft Corporation, Redmond, WA, USA) to extract the most relevant information from the selected articles and facilitate their subsequent analysis and interpretation. Study location, design, setting, main diagnosis, and analysis subgroups were extracted from each study. Subsequently, for each study and/or analysis subgroup we extracted: sample size, age, sex, functional level, left ventricular ejection fraction (LVEF), HF etiology, comorbidities, drug treatment, and cardiac devices and/or interventions.

The mean (SD) or median [IQR] score of the HRQoL questionnaire was extracted for each subgroup from studies that compared HRQoL based on the presence or not of non-cardiovascular comorbidities. In the case of studies that compared the prevalence of non-cardiovascular comorbidities based on the HRQoL score, the prevalence of patients with the studied non-cardiovascular comorbidity for each subgroup (better or worse HRQoL) was extracted. The meaning of the HRQoL score varied depending on the questionnaire used (Table 1). The statistical tests used to establish differences and/or associations were extracted for each study, as well as the p-values for the tests, considered significant or non-significant based on the established significance levels within each study, and odds ratios (OR) and 95% confidence intervals (CI) where available.

**Table 1:** Generic and specific questionnaires on health-related quality of life

Questionnaire	Dimensions	Punctuation
Generic		
EQ-5D	Mobility, personal care, daily activities, pain / discomfort and anxiety / depression	Index: from 0 (death) to 1 (best state), VAS: from 0 (worst state) to 100 (best state)
SF-36	Physical function, physical role, body pain, general health, vitality, social function, emotional role, mental health and declared evolution of health	Physical and mental summation: from 0 (worst state) to 100 (best state)
SF-12	Physical and mental	From 0 (worst functionality) to 100 (best functionality)
SF-6D	Physical function, role limitations, social function, pain, mental health and vitality	From 0 (death) to 1 (excellent health)
Specific		
KCCQ [50, 51]	Physical limitation, symptom stability, frequency of symptoms, severity of symptoms, self-care, quality of life and social limitation	Clinical summary (physical limitation and symptoms), summary of symptoms (frequency and severity) and general summary: from 0 (worst condition) to 100 (best condition)
MLHFQ [50, 51]	Physical, emotional, social	General: from 0 (best state) to 105 (worst state), physical component (0–40), and mental component (0–25)

*EQ-5D* European Quality of Life 5 Dimensions, *KCCQ* Kansas City Cardiomyopathy Questionnaire, *MLHFQ* Minnesota Living with Heart Failure Questionnaire, *SF-12* 12-item Short Form Health Survey, *SF-36* 36-item Short Form Health Survey, *SF-6D* Short Form Six Dimension

### Quality of studies included in the scoping review

We used the Quality Assessment Tool for Observational Cohort and Cross-Sectional Studies to assess the risk of bias at the level of study and outcome [25]. This tool consists of 14 items that allow subjectively assessing the quality of each article as poor, acceptable, or good: 1) relevance of the research question, 2) clear definition of the study population, 3) adequate population participation in study, 4) clarity and relevance of inclusion/exclusion criteria, 5) justification of the sample size, 6) measurement of exposure to the risk factor prior to inclusion in the study, 7) sufficient exposure time, 8) measurement of exposure by subgroups, 9) clarity in the definition of exposure measures, 10) adequate measurement of exposure over time, 11) clarity and relevance of outcome measures, 12) blinding of researchers to exposure, 13) patient follow-up rate, and 14) relevance and quality of statistical analyses.

### Data synthesis and presentation

Vote counting, based on the direction of the effect and statistical significance, was used to synthesize the findings from the present scoping review. For those studies that included HRQoL scores of patients with and without some associated non-cardiovascular comorbidity, relative differences were estimated. In addition, when the association between the presence of a comorbidity and the HRQoL was statistically significant, the direction of the association was indicated as “worse HRQoL” or “better HRQoL” to ease the interpretation of the results. Forest plots were further developed to provide a visual synthesis of the results. A meta-analysis could not be performed as no proper impact measure was consistently reported across all the studies included in the present scoping review.

## Results

### Study selection

**Fig. 1:**
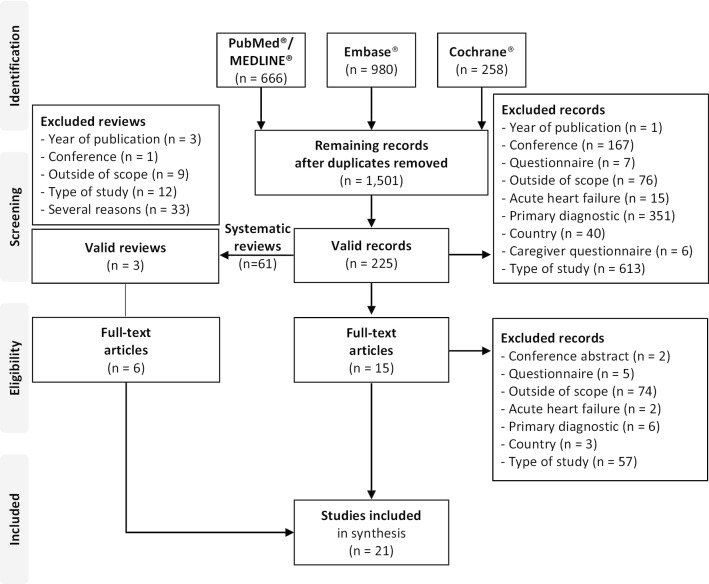
Flowchart of study selection

Figure 1 shows the flow diagram for this scoping review. The bibliographic search identified 1,904 records (666 in PubMed™/MEDLINE™, 980 in Embase™ and 258 in the Cochrane Database of Systematic Reviews), which were reduced to 1,501 articles after eliminating duplicates. Once screened by title and summary, the 225 articles that met the inclusion criteria were selected. Of these articles, after reviewing the full text, 15 original articles and 3 systematic reviews were selected. Finally, from the 3 systematic reviews, 6 articles were selected that met the inclusion criteria. Finally, 21 articles were selected [8, 14–18, 26–40]. Additional file 4 provides a list of included and excluded studies along with reasons for exclusion.

### Characteristics of the studies

Of the selected 21 articles (Table 2), 20 (95.2%) regarded observational studies [8, 14–18, 26, 27, 29–40]. Of these, 10 (50.0%) were cross-sectional studies [16–18, 30–34, 37, 38] and 10 (50.0%), cohort studies (9 prospective [8, 14, 15, 27, 29, 35, 36, 39, 40] and 1 retrospective [26]). In addition, an experimental study of which only baseline data was used for analysis was included [28]. Fifteen of the selected articles were multicenter studies (71.4%) [8, 15, 16, 26–30, 32, 33, 35–38, 40].

**Table 2:** Description of the selected studies

References	Location	Data source	Design	Timepoints used
Ancheta et al. 2009 [17]	USA	NA	SC, O, CS	NA
Arnold et al. 2016 [26]	USA	Interagency Registry for Mechanically Assisted Circulatory Support [52]	MC, O, C, R	12-month post LVAD
Bektas et al. 2017 [18]	The Netherlands	NA	SC, O, CS	NA
Bhatt et al. 2016 [27]	USA	The Atlanta Cardiomyopathy Consortium	MC, O, C, P	Baseline
Carson et al. 2009 [28]	USA	African-American Heart Failure Trial [53]	MC, E	Baseline
Chan et al. 2010 [29]	USA, Asia, Europe	Predictors of Response to Cardiac Re-Synchronization Therapy [54]	MC, O, C, P	6-month change
Comín-Colet et al. 2016 [30]	Spain	NA	MC, O, CS	NA
Comín-Colet et al. 2013 [14]	Spain	Unpublished data [14]	SC, O, C, P	Baseline
Cully et al. 2010 [31]	USA	NA	SC, O, CS	NA
Enjuanes et al. 2014 [32]	Poland, Spain, the Netherlands	The European Iron Consortium [55]	MC, O, CS	NA
Fotos et al. 2013 [16]	Greece	NA	MC, O, CS	NA
Fritschi and Redeker 2015 [33]	USA	Secondary analysis [56]	MC, O, CS	NA
Gastelurrutia et al. 2013 [34]	Spain	NA	SC, O, CS	NA
Harrow et al. 2011 [35]	USA	Women's Health Initiative for Health Utility Weights [57]	MC, O, C, P	Δ36m
Iqbal et al. 2010 [36]	UK	NA	MC, O, C, P	Baseline
Moliner et al. 2017 [37]	The Netherlands, Poland and Spain	The European Iron Consortium [55]	MC, O, CS	NA
Pantilant et al. 2016 [15]	USA	NA	MC, O, C, P	Baseline
Smolderen et al. 2009 [38]	The Netherlands	NA	MC, O, CS	NA
Staniute et al. 2015 [39]	Lithuania	NA	SC, O, C, P	Baseline
Streng et al. 2018 [8]	Europe	A Systems Biology Study to Tailored Treatment in Chronic Heart Failure [58]	MC, O, C, P	Baseline
Wienbergen et al. 2018 [40]	Germany and Switzerland	Registry Analysis of Iron Deficiency-Heart Failure (RAID-HF) [59]	MC, O, C, P	12-month / 12-month change

*C* cohort, *CS* cross-sectional, *E* experimental, *LVAD* left ventricular assist device, *MC* multi-center, *NA* not applicable, *O* observational, *P* prospective, *R* retrospective, *SC* single-center

### Characteristics of the participants

The patient samples included in the selected studies ranged between 96 and 3,499 patients and, when indicated, we found that they were mostly composed of non-hospitalized patients (11/15) [15, 17, 18, 27, 30, 31, 33, 34, 36–38], with a greater proportion of men than women (20/21) [8, 14–18, 26–34, 36–40], aged over 60 (16/20) [8, 14, 16–18, 26, 29–34, 36–38, 40], with New York Heart Association I-II functional level (10/18) [8, 14, 15, 18, 27, 30, 34, 36, 38, 39], and reduced LVEF (12/16) [8, 14, 17, 27–29, 32–34, 37, 38, 40] (Additional file 2: Tables 1 and 2).

In addition, 7 of the 10 articles that indicated the etiology identified ischemic heart disease as the main cause of HF [18, 30, 32, 34, 36, 37, 40]. Additionally, 2 articles identified a high prevalence of ischemic heart disease (40% of the sample), although they did not indicate other possible origins [14, 27]. One study found hypertensive heart disease to be the cause of HF [28] (Additional file 2: Table 3). The 20 articles that indicated the prevalence of comorbidities in the study sample recorded at least one comorbidity with a prevalence greater than 20% [8, 14–18, 26–34, 36–40], and 15 of them indicated the presence of multiple comorbidities, both cardiovascular and non-cardiovascular, with a prevalence greater than 20% [8, 14–16, 18, 27, 29, 30, 32, 34, 36–40] (Additional file 2: Table 4).

Additional file 2: Tables 5 and 6 show the treatments described in the articles [14–16, 18, 27–30, 32, 34, 36–38, 40], and the registered devices and interventions carried out [26, 27, 29, 38].

### Comorbidity and health-related quality of life

Among the selected articles, the relationship between 11 non-cardiovascular comorbidities of CHF (diabetes mellitus, mental and behavioral disorders, anemia, iron deficiency, respiratory system diseases, neoplasms, renal failure, arthritis, obesity, thyroid dysfunction, and hypercholesterolemia) and HRQoL was studied. HRQoL was measured exclusively through specific questionnaires in most cases (72.2%). The remaining studies measured HRQoL exclusively through generic questionnaires (16.7%), or through both generic and specific questionnaires (11.1%).

**Fig. 2:**
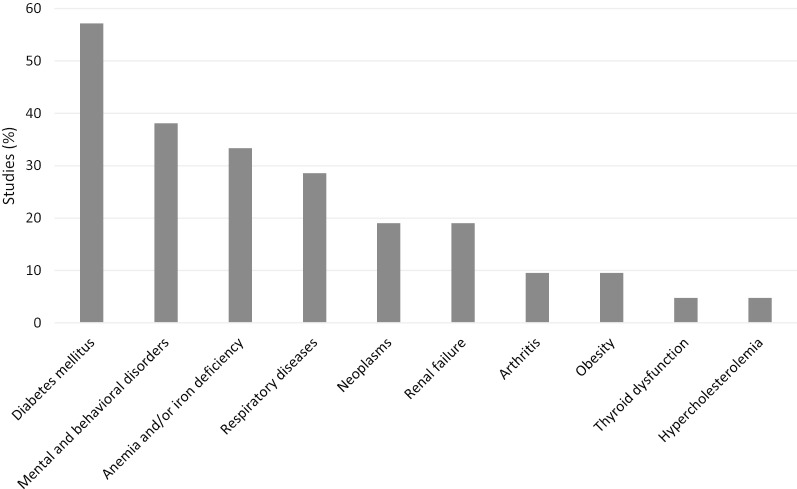
Articles that studied associations between non-cardiovascular comorbidities and health-related quality of life

The most common comorbidities studied were diabetes mellitus in 12 (57.1%) articles [8, 14, 16, 26, 28–30, 32, 33, 35, 36, 38], mental and behavioral disorders in 8 (38.1%) articles [15–17, 27, 31, 34, 36, 39], anemia and/or iron deficiency in 7 (33.3%) articles [8, 14, 30, 32, 35, 37, 40], and respiratory diseases in 6 (28.6%) articles [8, 16, 18, 26, 28, 36] (Fig. 2).

**Fig. 3:**
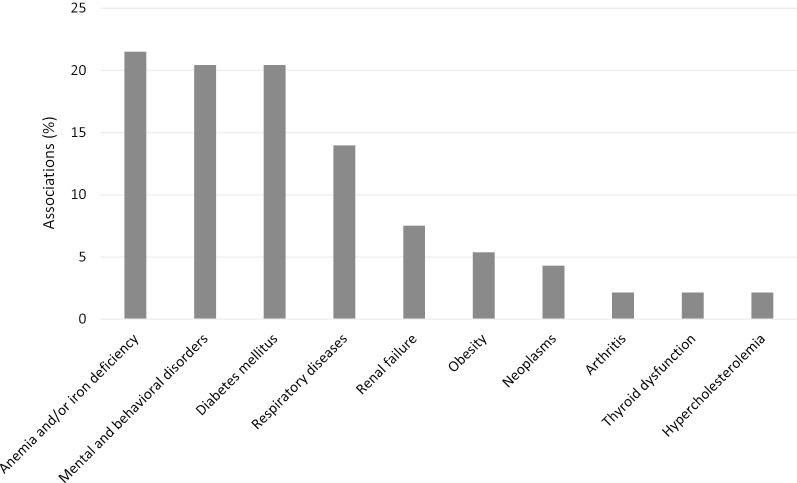
Associations studied between non-cardiovascular comorbidities and health-related quality of life

**Fig. 4:**
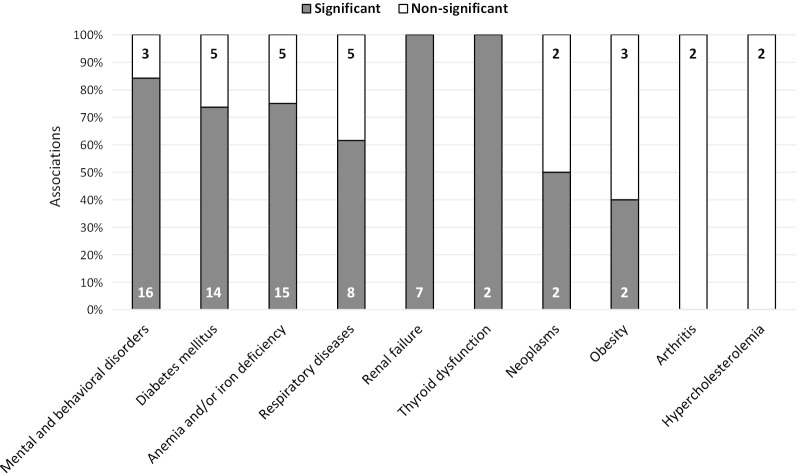
Proportion of significant and non-significant associations between non-cardiovascular comorbidities and worse health-related quality of life. Numbers are indicative of the number of significant or non-significant associations

In total, 93 possible associations between different non-cardiovascular comorbidities and HRQoL were tested across studies. Most of these associations regarded anemia or iron deficiency (21.5%, 7 articles) [8, 14, 30, 32, 35, 37, 40], followed by mental and behavioral disorders (20.4%, 8 articles) [15–17, 27, 31, 34, 36, 39], diabetes mellitus (20.4%, 12 articles) [8, 14, 16, 26, 28–30, 32, 33, 35, 36, 38] and respiratory diseases (14.0%, 6 articles) [8, 16, 18, 26, 28, 36] (Fig. 3). Some of these associations were analyzed with different statistical tests, and most indicated a statistically significant relationship between the presence of comorbidity and a worse HRQoL (Fig. 4). Although less studied, statistically significant associations were also observed between the presence of renal failure [8, 14, 16, 30], thyroid gland dysfunction [8], neoplasms [16, 26, 35, 36] or obesity [8, 30], and a worse HRQoL in patients with HF (Fig. 4).

**Fig. 5:**
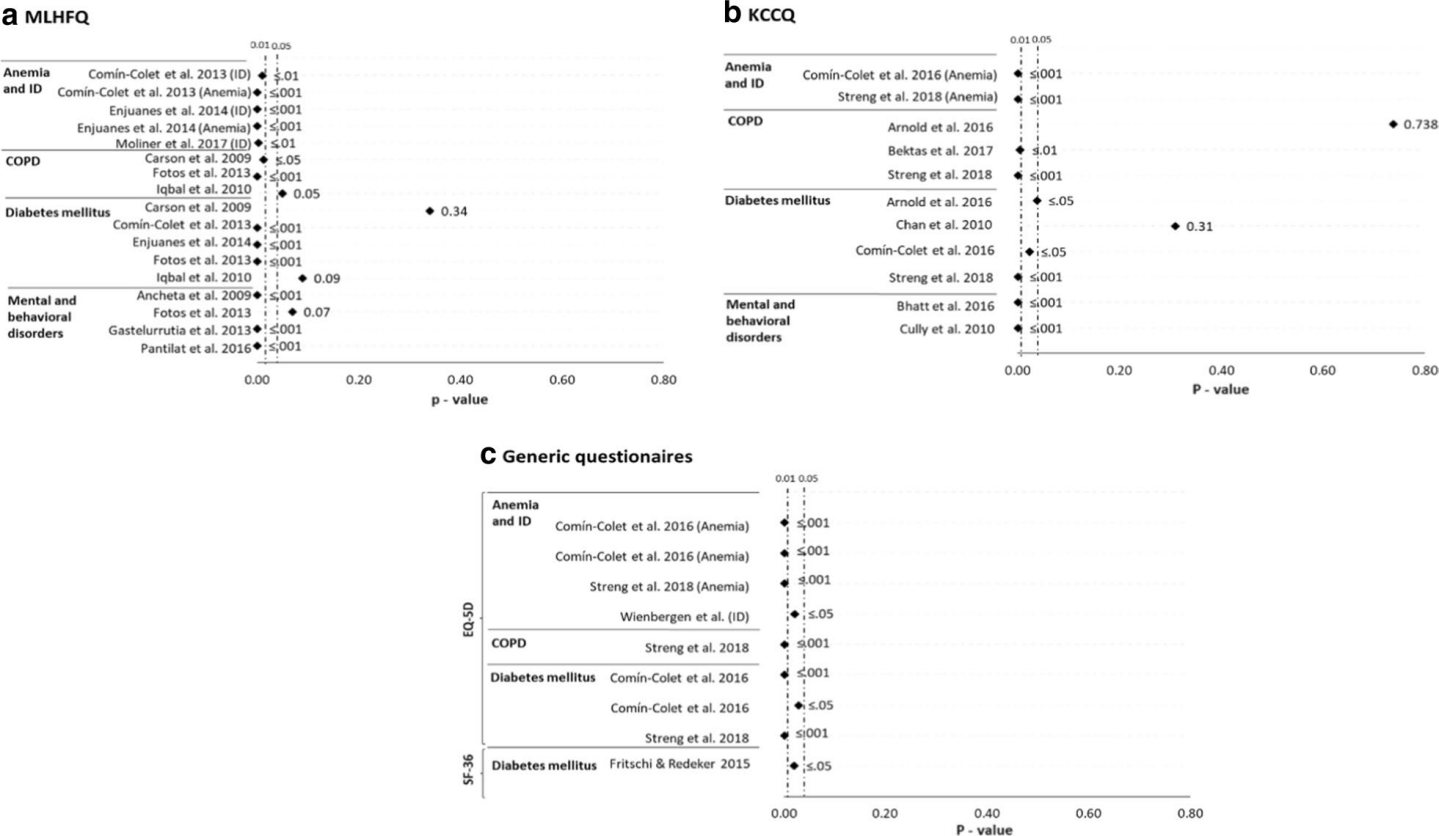
Statistical significance of the associations between non-cardiovascular comorbidities and worse health-related quality of life. Non-cardiovascular comorbidities are anemia or iron deficiency [ID], chronic obstructive pulmonary disease [COPD], diabetes mellitus, and mental and behavioral disorders. Health-related quality of life is according to the Minnesota Living with Heart Failure Questionnaire (MLHFQ) (**a**), the Kansas City Cardiomyopathy Questionnaire (KCCQ) (**b**), and the generic European Quality of Life 5 Dimensions (EQ-5D) and 36-item Short Form Health survey (SF-36) (**c**). The associations represented are those in which the health-related quality of life was valued globally

**Fig. 6:**
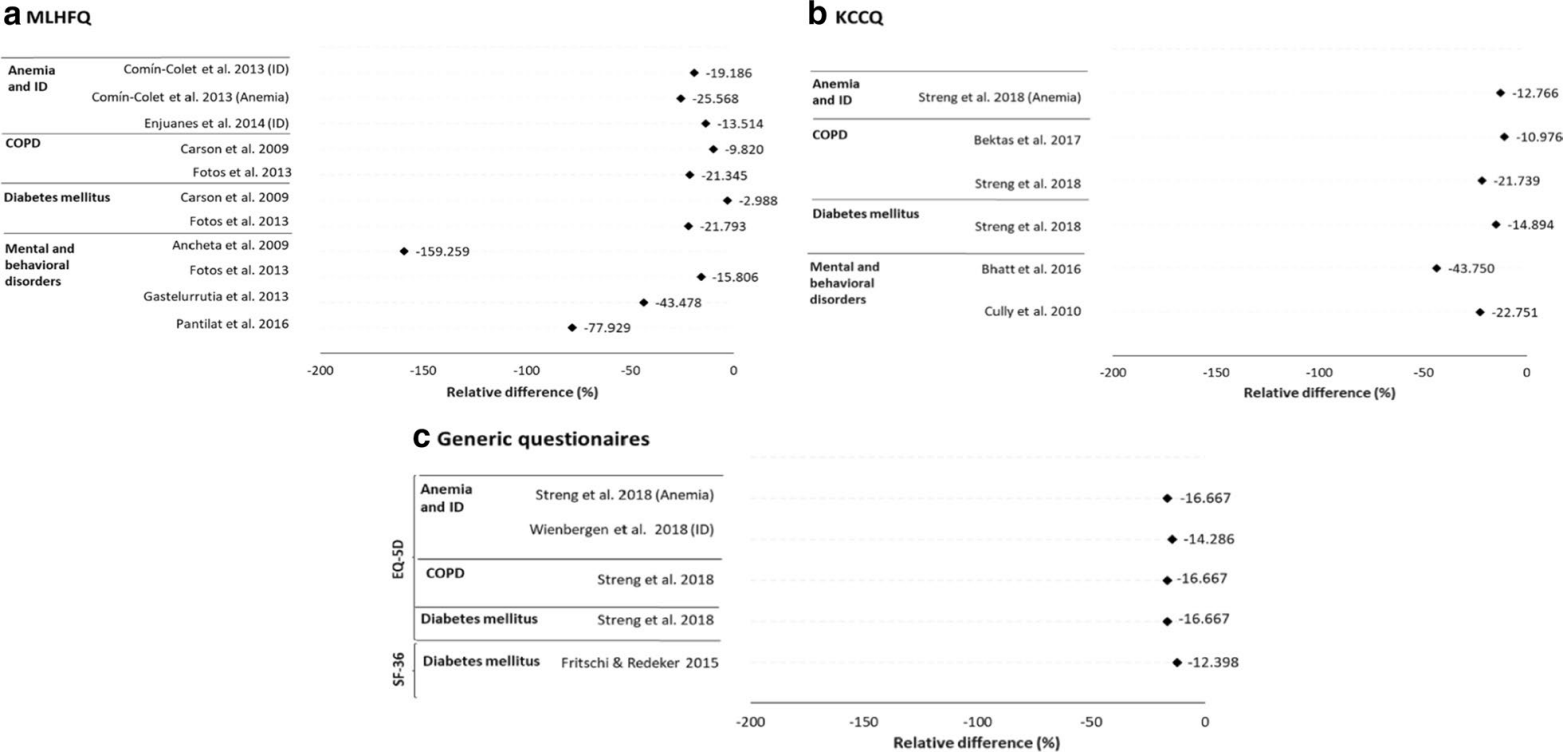
Relative health-related quality of life of patients with non-cardiovascular comorbidities compared to patients without comorbidities. Non-cardiovascular comorbidities are anemia or iron deficiency (ID), chronic obstructive pulmonary disease (COPD), diabetes mellitus, or mental and behavioral disorders. Health-related quality of life is according to the Minnesota Living with Heart Failure Questionnaire (MLHFQ) (**a**), the Kansas City Cardiomyopathy Questionnaire (KCCQ) (**b**), and the generic European Quality of Life 5 Dimensions (EQ-5D) and 36-item Short Form Health survey (SF-36) (**c**). The differences represented are those in which the health-related quality of life was valued globally. The sign of relative health-related quality of life in the MLHFQ has been modified in such a way that the negative difference means a worse quality of life for patients with comorbidities

Non-cardiovascular comorbidities with the greatest negative impact on HRQoL are, in order of importance: anemia and iron deficiency, mental and behavioral disorders, diabetes mellitus, and respiratory diseases. Figure 5 shows a large proportion of the associations between one of these four comorbidities and a worse HRQoL were statistically significant. The values represented in this figure are those of the univariate regression analyses, due to their easier comparison. However, most of the associations tested by multivariate regressions were also significant. In all significant associations, the impact was always negative regardless of the type of questionnaire used to measure HRQoL (Fig. 6).

#### Anemia and iron deficiency

A statistically significant and negative impact on HRQoL was found with anemia or iron deficiency in 75.0% (15/20) of the associations studied [8, 14, 30, 32, 37, 40] (Fig. 4). More than half of the associations (11/20) were studied using the specific MLHFQ [14, 32, 37], with 8 being statistically significant (72.7%) [14, 32, 37]. One of these studies reported that patients with both anemia and iron deficiency were 2.2 times more likely to have a worse HRQoL than patients without anemia or iron deficiency (95% CI 1.3–3.6; *p* = 0.002) [32]. Moreover, patients with iron deficiency who had not developed anemia were 1.6 times more likely to have a worse HRQoL than patients without anemia or iron deficiency (95% CI 1.1–2.6; *p* = 0.025) [32]. However, the HRQoL of patients with anemia and without iron deficiency did not differ from that of patients without anemia or iron deficiency [32]. If we take into account anemia and iron deficiency separately, of the 20 associations studied, 10 analyzed anemia [8, 14, 30, 32, 35] and 10 analyzed iron deficiency as comorbidities [14, 32, 37, 40].

The association between anemia and HRQoL was statistically significant in 8 out of 10 cases (80.0%) [8, 14, 30, 32]. The 3 associations studied when measuring HRQoL with the generic EQ-5D questionnaire were found to be significant [8, 30].Moreover, the association between iron deficiency and HRQoL was statistically significant in 7 out of 10 cases (70.0%) [14, 32, 37, 40], 5 of them using the MLHFQ [14, 32, 37]. The presence of other comorbidities within multivariate analyses performed was common for all non-significant associations observed between anemia and worse HRQoL [14, 32, 35], while the only multivariate analysis that found a significant association between anemia and worse HRQoL did not adjust for other comorbidities [8]. One study aimed to determine the impact of impaired iron storage (IIS) or transport (IIT) with respect to a normal iron status (NIS) and found that patients with both IIS and IIT were 1.8 times more likely to have a worse HRQoL than patient with NIS (95% CI 1.2–2.7; *p* = 0.003) [37]. Moreover, patients with isolated IIT were 1.7 times more likely to have a worse HRQoL than patient with NIS (95% CI 1.2–2.5; *p* = 0.005) [37]. However, the HRQoL of patients with isolated IIS did not differ from that of patients with NIS [37]. Additional file 2: Table 7 shows these results in detail.

#### Mental and behavioral disorders

Most of the associations studied between mental and behavioral disorders, and HRQoL (84.2%, 16/19) found mental and behavioral disorders, mainly depression, had a statistically significant and negative impact on HRQoL [15–17, 27, 31, 34, 36, 39] (Fig. 4). These associations were studied exclusively with the specific questionnaires KCCQ or MLHFQ. Using the KCCQ, we observed that patients with depression obtained worse overall HRQoL scores than those without depression in a range of 22.8–43.8% [27, 31]. Moreover, patients with depression were 60.8 times more likely to have a poor HRQoL compared to patients with minimal symptoms of depression (95% CI 18.1–204; *p* < 0.001), and even patients with mild symptoms of depression were 13.4 times more likely to have a poor HRQoL compared to patients with minimal symptoms of depression (95% CI 4.13–43.7; *p* < 0.001) [27]. With the MLHFQ, we found that patients with depression obtained worse overall HRQoL scores than those without depression in a range of 43.5–159.3% [15, 17, 34]. One of these studies assessed patients at a 3-month follow-up and found that those with a clinically meaningful improvement in their depression scores were 4.3 times more likely to report a clinically meaningful improvement in their HRQoL (95% CI 1.2–14.6; *p* = 0.002) [15]. Additional file 2: Table 8 shows these results in detail.

#### Diabetes mellitus

Most studies (73.7%, 14/19) found diabetes mellitus had a statistically significant impact on HRQoL [8, 14, 16, 26, 30, 32, 33, 38] (Fig. 4); 36.8% (7/19) of the associations were studied using the MLHFQ [14, 16, 28, 32, 36], of which 5 were statistically significant (71.4%) [14, 16, 32]. The only study that provided the overall HRQoL score using the MLHFQ observed a significantly worse score patients with diabetes mellitus compared to those without this comorbidity (21.8%) [16]. Additional file 2: Table 9 shows these results in detail.

#### Diseases of the respiratory system

A negative and statistically significant relationship was found in most (61.5%, 8/13) of the studies between respiratory diseases, mainly chronic obstructive pulmonary disease (COPD), and HRQoL [8, 16, 18, 28] (Fig. 4). The KCCQ was used in 69.2% (9/13) of the associations [8, 18, 26], of which 55.6% (5 associations) were statistically significant [8, 18]. Patients with COPD obtained significantly worse overall HRQoL scores than those without COPD in a range of 11.0–21.7% [8, 18]. Additional file 2: Table 10 shows these results in detail.

### Quality of the selected studies

In general, the selected studies were of acceptable quality, although many did not provide sufficient information about threats to internal validity. However, 33.3% (7 of 21) of the studies were considered to be of good quality [15, 26, 27, 29, 35, 39, 40]. These studies stood out for their transparency and control of threats to internal validity. Additional file 3 shows the results of this assessment in detail.

## Discussion

The objective of this scoping review was to identify and describe the available evidence on the impact that non-cardiovascular comorbidities have on the HRQoL of patients with CHF. In total, 21 articles met the inclusion criteria. Our results indicate that the presence of a non-cardiovascular comorbidity has a negative and, in almost all cases, statistically significant impact on the HRQoL of these patients, which is also independent of other non-cardiovascular comorbidities. This finding stayed true for all diseases whose association with HRQoL was studied, regardless of study design, type of questionnaire used to measure HRQoL, and analysis method.

Among the studies that met the inclusion criteria of this review, we found that most of the estimated associations between a non-cardiovascular disease and HRQoL correspond to four main non-cardiovascular disease groups: anemia and iron deficiency (21.5% of the total associations studied, in 7 articles), respiratory diseases (mainly COPD, with 14.0% of the total associations studied, in 6 articles), diabetes mellitus (20.4% of the associations, in 12 articles), and mental and behavioral disorders (20.4% of the associations, in 8 articles). All the associations studied in these four disease groups showed a negative impact between the non-cardiovascular comorbidity and HRQoL, being statistically significant in 74.6% of cases. Statistical significance was found in 61.5%, 73.7% and 84.2% of the associations that studied the impact of COPD, diabetes mellitus, and mental and behavioral disorders on HRQoL, respectively. In the case of anemia and iron deficiency, the group with most measures of impact analyzed, 75% of the associations were statistically significant. Studies that focused on anemia and iron deficiency in the same sample observed a significant association using comparative non-adjusted analyses. However, after controlling for covariates, only iron deficiency remained significantly associated with worse HRQoL in all studies.

Although not as frequently studied in the selected articles, a significant association was also observed between renal insufficiency, thyroid dysfunction, malignancies, and obesity and worse HRQoL in patients with CHF. Overall, the quality of the studies reviewed was considered acceptable.

The results of this scoping review are of significant relevance, providing the basis for future research to guide clinical practice on this matter, since they highlight the importance of treating comorbidities in the overall management of patients with CHF in order to improve their HRQoL. In this context, the key focus should be placed on improving the HRQoL of patients with CHF so that the comorbidities are also targeted and not only the CHF. Previous studies on the effect of multimorbidity on the HRQoL have arrived at similar conclusions. For example, diabetic patients with no other vascular risk factors or vascular disease have a similar HRQoL to that of the general non-diabetic population, after adjusting for covariates. However, when the diabetic patient has several risk factors or has developed vascular disease, the HRQoL decreases significantly [41].

The importance of treating comorbidities in the integrated management of patients with CHF has been evidenced in the guide for the diagnosis and treatment of patients with CHF developed by the European Society of Cardiology in 2016 [2]. However, given that some comorbidities have a higher prevalence than others, these should be prioritized when caring for the patient. The measures of impact that were more frequently studied in the articles included in this review were anemia and iron deficiency. Previous studies show that iron deficiency has a prevalence of 53–65% in patients with CHF [13, 42], and is responsible for at least 50% of cases of anemia [43]. In addition, several studies have identified a high rate of under diagnosis of iron deficiency [44–46]. These findings, together with the those of our review, show that iron deficiency and anemia are key and independent factors that contribute more strongly to the worsening of the HRQoL of patients with CHF than other widely studied non-cardiovascular comorbidities, such as diabetes and COPD. This is, although the impact of each non-cardiovascular comorbidity on the HRQoL of patients with CHF is comparable, anemia and iron deficiency become more relevant given their higher prevalence in these patients. This prevalence has a significant impact on the global epidemiological burden of CHF, and its treatment is key in the improvement of HRQoL in patients with CHF. In this regard, it has been observed that patients with CHF and iron deficiency can benefit from intravenous treatment with ferric carboxymaltose [47, 48], reaching a HRQoL at least similar to that of patients with CHF without this comorbidity [8, 40]. Further studies that highlight the improvement of the HRQoL by treating comorbidities in CHF are necessary.

The integrated management of CHF from its diagnosis and that of associated non-cardiovascular comorbidities is relevant in the context of clinical practice to increase the HRQoL of patients but it can also be essential in reducing the economic burden on society [49]. Indeed, non-cardiovascular comorbidities, such as anemia, diabetes, or chronic renal failure, greatly increase the risk of hospitalization, length of hospital stay, and mortality of patients with CHF compared to those without these comorbidities [5]. Specifically, patients with CHF with five or more comorbidities account for 81% of hospitalization days of all patients with CHF [7]. On this note, there has been an increase in hospitalizations of patients with CHF due to non-cardiovascular causes compared to those due to cardiovascular causes [11]. Likewise, an increase in the diagnosis of non-cardiovascular comorbidities has been observed in patients hospitalized with CHF [12], suggesting that many of these hospitalizations could be due to the presence of non-cardiovascular comorbidities. We have not found studies assessing the direct associated hospital resource cost due to CHF-associated comorbidities, but we cannot rule out the possibility of it being significant. Likewise, the association of non-cardiovascular comorbidities with higher rates of hospitalization and length of hospital stay could lead to other significant social costs (job loss, professional care, informal care) for patients and society in general. Comprehensive patient management could reduce these costs for healthcare systems and society, and that future studies that estimate the costs associated with non-cardiovascular comorbidities in patients with CHF are warranted.

Several limitations of this review should be taken into account when evaluating its results. First, we did not publish a protocol of the study prior to running the scoping review, and the methods were therefore not peer-reviewed prior to our search. Second, although English is the language of the vast majority of scientific journals, including non-English language studies or using additional search engines could have led to a greater number of studies selected for the review. Third, we could not perform a meta-analysis due to the heterogeneity of the experimental designs, samples, comorbidity assessment tools, and HRQoL assessment questionnaires used in the selected studies. Fourth, most studies were not designed to respond to the objective of this review, so there are limitations inherent to the observational and cross-sectional designs, threats to the internal validity of the selected studies, lack of information such as severity of comorbidities and how this could affect the impact of HRQoL, and cases in which different results were obtained based on the statistical test used. However, observational designs allowed studying the presence of associated comorbidities that would have been part of the exclusion criteria in another type of study. In addition, given the multicenter design of most of these studies, the results could be generalized to the rest of the population with CHF.

## Conclusions

The results of this scoping review show that the impact of non-cardiovascular comorbidities on the HRQoL of patients with CHF is consistently negative and significant. The comorbidities whose impact has been studied most frequently in the literature are anemia and iron deficiency, respiratory diseases, mental disorders, and diabetes. The presence of comorbidities whose impact on HRQoL has been less studied (renal insufficiency, thyroid dysfunction, neoplasms, obesity) also showed a significant association with a worse HRQoL.

The results highlight the relevance of including the comorbidities associated with a worse HRQoL in the integrated clinical management of CHF, with special emphasis on treating or adequately controlling comorbidities of greater prevalence in these patients. Control of these comorbidities can contribute not only to significantly increasing the HRQoL of patients but it can also provide other social benefits. Further studies are needed to clearly estimate the impact that non-cardiovascular comorbidities have on HRQoL in patients with CHF.

## Supplementary information

**Additional file 1:** Search strategies performed on each database. Description of the complete search strategy on each database.

**Additional file 2:** Summary of individual studies. Detailed summary of individual studies

**Additional file 3:** Risk of bias for individual studies. Risk of bias grading for individual studies according to the Quality Assessment Tool for Observational Cohort and Cross-Sectional Studies

**Additional file 4:** List of included and excluded studies. Detailed list of included and excluded studies by title and/or abstract and full-text records with reasons for exclusion.

## Data Availability

The datasets supporting the conclusions of this article are included within the article and its additional files.

## Abbreviations

C: Cohort
CHF: Chronic heart failure
COPD: Chronic obstructive pulmonary disease
CS: Cross-sectional
E: Experimental
EQ-5D: European Quality of Life-5 Dimensions
HF: Heart failure
HRQoL: Health-related quality of life
ID: Iron deficiency
KCCQ: Kansas City Cardiomyopathy Questionnaire
LVAD: Left ventricular assist device
LVEF: Left ventricular ejection fraction
MC: Multi-center
MLHFQ: Minnesota Living with Heart Failure Questionnaire
NA: Not applicable
O: Observational
P: Prospective
R: Retrospective
SC: Single-center
SF-12: 12-Item Short Form Health Survey
SF-36: 36-Item Short Form Health Survey
SF-6D: Short Form Six Dimension
