# Supplementary material for: Impact of non-cardiovascular comorbidities on the quality of life of patients with chronic heart failure: a scoping review
Source: Health Qual Life Outcomes. 2020 Oct 7;18:329. doi: 10.1186/s12955-020-01566-y (PMC7542693; doi:10.1186/s12955-020-01566-y)
Supplement: Supplementary file 1 — Additional file 1: Search strategies performed on each database. Description of the complete search strategy on each database. [file 12955_2020_1566_MOESM1_ESM.docx]

**Additional file 1** Search strategies performed on each database

**Search strategy on PubMed®/MEDLINE®**

(heart failure[Title/Abstract] OR backward failure, heart[Title/Abstract] OR cardiac backward failure[Title/Abstract] OR cardiac decompensation[Title/Abstract] OR cardiac failure[Title/Abstract] OR cardiac incompetence[Title/Abstract] OR cardiac insufficiency[Title/Abstract] OR cardiac stand still[Title/Abstract] OR cardiac decompensation[Title/Abstract] OR cardiac insufficiency[Title/Abstract] OR chronic heart failure[Title/Abstract] OR chronic heart insufficiency[Title/Abstract] OR decompensation cordis[Title/Abstract] OR decompensation,heart[Title/Abstract] OR heart backward failure[Title/Abstract] OR heart decompensation[Title/Abstract] OR heart failure[Title/Abstract] OR heart incompetence[Title/Abstract] OR heart insufficiency[Title/Abstract] OR insufficient cardis[Title/Abstract] OR myocardial failure[Title/Abstract] OR myocardial insufficiency[Title/Abstract]) AND (quality of life[Title/Abstract] OR hrql[Title/Abstract] OR health related quality of life[Title/Abstract] OR life quality[Title/Abstract] OR quality of life[Title/Abstract] OR patient reported outcome[Title/Abstract] OR patient reported outcome measures[Title/Abstract] OR patient reported outcome[Title/Abstract] OR patient reported treatment outcome[Title/Abstract] OR patient reported outcome[Title/Abstract] OR self reported outcome[Title/Abstract] OR self reported patient outcome[Title/Abstract] OR self reported treatment outcome[Title/Abstract] OR self reported outcome[Title/Abstract] OR health care survey[Title/Abstract] OR health care survey[Title/Abstract] OR health care surveys[Title/Abstract] OR healthcare survey[Title/Abstract] OR health status[Title/Abstract] OR clinical state[Title/Abstract] OR health state[Title/Abstract] OR health status[Title/Abstract] OR minnesota living with heart failure questionnaire[Title/Abstract] OR minnesota living with heart failure questionnaire[Title/Abstract] OR mlhfq[Title/Abstract] OR kansas city cardiomyopathy questionnaire[Title/Abstract] OR kansas city cardiomyopathy questionnaire[Title/Abstract] OR kccq[Title/Abstract] OR european quality of life-5 dimensions[Title/Abstract] OR euroqol 5d[Title/Abstract] OR eq 5d[Title/Abstract] OR short form 36[Title/Abstract] OR 36 item short form health survey[Title/Abstract] OR sf-36[Title/Abstract] OR sf36[Title/Abstract] OR short form 36[Title/Abstract] OR short form 36 health survey[Title/Abstract] OR sf 6d[Title/Abstract] OR the health utilities index[Title/Abstract]) Filters: Observational Study; Systematic Reviews; Multicenter Study; Humans; English; Publication date from 2009/01/01 to 2018/12/31

**Search strategy on Embase®**

('backward failure, heart':ab,ti OR 'cardiac backward failure':ab,ti OR 'cardiac failure':ab,ti OR 'cardiac incompetence':ab,ti OR 'cardiac stand still':ab,ti OR 'cardiac decompensation':ab,ti OR 'cardiac insufficiency':ab,ti OR 'chronic heart failure':ab,ti OR 'chronic heart insufficiency':ab,ti OR 'decompensation cordis':ab,ti OR decompensation,heart:ab,ti OR 'heart backward failure':ab,ti OR 'heart decompensation':ab,ti OR 'heart failure':ab,ti OR 'heart incompetence':ab,ti OR 'heart insufficiency':ab,ti OR 'insufficient cardis':ab,ti OR 'myocardial failure':ab,ti OR 'myocardial insufficiency':ab,ti) AND (hrql:ab,ti OR 'health related quality of life':ab,ti OR 'life quality':ab,ti OR 'quality of life':ab,ti OR 'patient reported outcome measures':ab,ti OR 'patient reported treatment outcome':ab,ti OR 'patient reported outcome':ab,ti OR 'self reported patient outcome':ab,ti OR 'self reported treatment outcome':ab,ti OR 'self reported outcome':ab,ti OR 'health care survey':ab,ti OR 'health care surveys':ab,ti OR 'healthcare survey':ab,ti OR 'clinical state':ab,ti OR 'health state':ab,ti OR 'health status':ab,ti OR 'minnesota living with heart failure questionnaire':ab,ti OR mlhfq:ab,ti OR 'kansas city cardiomyopathy questionnaire':ab,ti OR kccq:ab,ti OR 'european quality of life-5 dimensions':ab,ti OR 'euroqol 5d':ab,ti OR 'eq 5d':ab,ti OR '36 item short form health survey':ab,ti OR 'sf 36':ab,ti OR sf36:ab,ti OR 'short form 36':ab,ti OR 'short form 36 health survey':ab,ti OR 'sf 6d':ab,ti OR 'the health utilities index':ab,ti) AND ('multicenter study'/de OR 'observational study'/de OR 'systematic review'/de) AND [english]/lim AND [humans]/lim AND [1-1-2009]/sd NOT [1-1-2019]/sd

**Search strategy on the Cochrane Database of Systematic Reviews**

heart failure OR backward failure, heart OR cardiac backward failure OR cardiac decompensation OR cardiac failure OR cardiac incompetence OR cardiac insufficiency OR cardiac stand still OR cardiac decompensation OR cardiac insufficiency OR chronic heart failure OR chronic heart insufficiency OR decompensation cordis OR decompensation,heart OR heart backward failure OR heart decompensation OR heart failure OR heart incompetence OR heart insufficiency OR insufficient cardis OR myocardial failure OR myocardial insufficiency in Title Abstract Keyword AND quality of life OR hrql OR health related quality of life OR life quality OR quality of life OR patient reported outcome OR patient reported outcome measures OR patient reported outcome OR patient reported treatment outcome OR patient reported outcome OR self reported outcome OR self reported patient outcome OR self reported treatment outcome OR self reported outcome OR health care survey OR health care survey OR health care surveys OR healthcare survey OR health status OR clinical state OR health state OR health status OR minnesota living with heart failure questionnaire OR minnesota living with heart failure questionnaire OR mlhfq OR kansas city cardiomyopathy questionnaire OR kansas city cardiomyopathy questionnaire OR kccq OR european quality of life-5 dimensions OR euroqol 5d OR eq 5d OR short form 36 OR 36 item short form health survey OR sf-36 OR sf36 OR short form 36 OR short form 36 health survey OR sf 6d OR the health utilities index in Title Abstract Keyword - with Cochrane Library publication date Between Jan 2009 and Dec 2018, in Cochrane Reviews (Word variations have been searched)
