# Supplementary material for: Impact of non-cardiovascular comorbidities on the quality of life of patients with chronic heart failure: a scoping review
Source: Health Qual Life Outcomes. 2020 Oct 7;18:329. doi: 10.1186/s12955-020-01566-y (PMC7542693; doi:10.1186/s12955-020-01566-y)
Supplement: Supplementary file 2 — Additional file 2: Summary of individual studies. Detailed summary of individual studies [file 12955_2020_1566_MOESM2_ESM.docx]

**Additional file 2** Summary of individual studies

**Table 1.** Overview of participants and comparative groups

|  | **Setting** | **Heart failure diagnosis** | **Groups** | **Definition** |
| --- | --- | --- | --- | --- |
| Ancheta et al. 2009 [17] | Outpatient | HF with reduced EF, NYHA II-III | Total cohort | Total cohort |
| Arnold et al. 2016 [26] |  | End-stage, CHF + LVAD indication | Poor outcome | Death or mean KCCQ SS <45 throughout one-year post LVAD |
|  |  |  | Acceptable outcome | One-year survival post LVAD with mean KCCQ SS ≥45 |
| Bektas et al., 2017 [18] | Outpatient | CHF with LVD, NYHA I-IV | Total cohort | Total cohort |
|  |  |  | With COPD | FEV1/FVC <0.70 after bronchodilatation |
|  |  |  | Without COPD | Without COPD |
| Bhatt et al. 2016 [27] | Outpatient | HF with LVD | Total cohort | Total cohort |
|  |  |  | Minimal depressive symptoms | PHQ-9 score 0–4 |
|  |  |  | Mild depressive symptoms | PHQ-9 score 5–9 |
|  |  |  | Depression | PHQ-9 score 10–27 |
| Carson et al. 2009 [28] |  | CHF with LVD, NYHA III-IV | Total cohort | Total cohort |
| Chan et al. 2010 [29] |  | HF with reduced EF, NYHA III-IV | Total cohort | Total cohort |
| Comín-Colet at al. 2016 [30] | Outpatient | CHF with reduced EF, NYHA I-IV | Total cohort | Total cohort |
|  |  |  | Better HRQoL | KCCQ ≥50, EQ-5D index ≥0.5, EQ-5D VAS ≥50 |
|  |  |  | Worse HRQoL | KCCQ <50, EQ-5D index <0.5, EQ-5D VAS <50 |
| Comín-Colet et al. 2013 [14] |  | CHF with reduced/preserved EF, NYHA I-IV | Total cohort | Total cohort |
|  |  |  | With ID | Ferritin (ng/mL): <100 or <800 and TSAT <20% |
|  |  |  | Without ID | Without ID |
| Cully et al. 2010 [31] | Outpatient | HF, NYHA II-IV | Total cohort | Total cohort |
|  |  |  | Symptomatic depression | GDS ≥6 |
|  |  |  | Non-symptomatic depression | GDS <6 |
| Enjuanes et al. 2014 [32] |  | CHF with reduced/preserved EF, NYHA I-IV | Total cohort | Total cohort |
|  |  |  | With ID | Ferritin (µg/mL): <100 or 100–299 and TSAT <20% |
|  |  |  | Without ID | Without ID |
| Fotos et al. 2013 [16] | Inpatient | HF, NYHA III-IV | Total cohort | Total cohort |
| Fritschi & Redeker 2015 [33] | Outpatient | CHF, NYHA I-IV | Total cohort | Total cohort |
|  |  |  | With DM | Report of DM |
|  |  |  | Without DM | Report of DM |
| Gastelurrutia et al. 2013 [34] | Outpatient | CHF, NYHA I-IV | Total cohort | Total cohort |
| Harrow et al. 2011 [35] |  | Self-identified HF | Total cohort | Total cohort |
| Iqbal et al. 2010 [36] | Outpatient/discharge | CHF with LVD, NYHA I-IV | Total cohort | Total cohort |
|  |  |  | Good HRQoL | MLHFQ < Median |
|  |  |  | Poor HRQoL | MLHFQ ≥ Median |
| Moliner et al. 2017 [37] | Outpatient | CHF with reduced/preserved EF, NYHA I-IV | IIS | Ferritin <100µg/mL and TSAT ≥20% |
|  |  |  | IIT | Ferritin ≥100µg/mL and TSAT <20% |
|  |  |  | IIS+IIT | Ferritin <100µg/mL and TSAT <20% |
|  |  |  | NIS | Without IIS and/or IIT |
| Pantilant et al. 2016 [15] | Outpatient | HF with reduced/preserved EF, NYHA II-III | Total cohort | Total cohort |
| Smolderen et al. 2009 [38] | Outpatient | CHF with reduced EF, NYHA I-III | Total cohort | Total cohort |
| Staniute et al. 2015 [39] | Inpatient | CAD + HF | Total cohort | Total cohort |
| Streng et al. 2018 [8] | In/outpatient | CHF with reduced/preserved EF, NYHA I-IV | Total cohort | Total cohort |
|  |  |  | Reduced EF | EF <40% |
|  |  |  | Mid-range EF | EF 40-49% |
|  |  |  | Preserved EF | EF ≥50% |
| Wienbergen et al. 2018 [40] | In/outpatient | CHF with reduced EF, NYHA I-IV | With ID | Ferritin (µg/mL) <100 or 100–299 and TSAT<20% |
|  |  |  | Without ID | Without ID |

CAD (coronary artery disease), CHF (chronic heart failure) COPD (chronic obstructive pulmonary disease), DM (diabetes mellitus), EF (ejection fraction), EQ-5D (EuroQoL 5 dimensions), FEV1 (one-second forced expiratory volume), FVC (forced vital capacity), GDS (Geriatric Depression Scale), HRQoL (health-related quality of life), ID (iron deficiency), IIS (impaired iron storage), IIT (impaired iron transport), KCCQ SS (Kansas City Cardiomyopathy Questionnaire Summary Score), LVAD (left ventricular assist device), LVD (left ventricular dysfunction), MLHFQ (Minnesota Living with Heart Failure Questionnaire), NIS (normal iron status), NYHA (New York Heart Association), PHQ-9 (Patient Health Questionnaire-9), TSAT (transferrin saturation), VAS (visual analogue scale). Missing information accounts for a lack of data availability within selected studies.

**Table 2.** Patient samples in studies

|  | **Groups** | **Baseline (n)** | **Age (years)** | | **F (%)** | **M (%)** | **NYHA class** | | **NYHA**  **I-II (%)** | **NYHA**  **III-IV (%)** | **LVEF (%)** | | **Reduced EF or LVEF <40% (%)** | **Preserved EF or LVEF ≥50% (%)** | **Mid-range EF or LVEF 40–49% (%)** |  |
| --- | --- | --- | --- | --- | --- | --- | --- | --- | --- | --- | --- | --- | --- | --- | --- | --- |
| Ancheta et al. 2009 [17] | Total cohort | 108 | 64.9 | (12.3) | 44.0 | 56.0 |  |  | 30.0 | 70.0 | 26.5 | (8.2) | 100.0 | 0.0 | 0.0 |  |
| Arnold et al. 2016 [26] | Poor outcome |  |  |  | 18.6 | 81.4 |  |  |  |  |  |  |  |  |  | *a* |
|  | Acceptable outcome |  |  |  | 18.8 | 81.2 |  |  |  |  |  |  |  |  |  |  |
| Bektas et al., 2017 [18] | Total cohort | 186 | 76.0 | [68-82] | 31.0 | 69.0 |  |  | 78.0 | 21.0 | 44.0 | [32-54] |  | 31.0 |  |  |
|  | With COPD | 118 | 76.0 | [67-82] | 34.0 | 66.0 |  |  | 84.0 | 16.0 | 46.0 | [32-57] |  | 38.0 |  |  |
|  | Without COPD | 68 | 76.0 | [69-84] | 25.0 | 75.0 |  |  | 69.0 | 31.0 | 43.0 | [30-50] |  | 27.0 |  |  |
| Bhatt et al. 2016 [27] | Total cohort | 308 | 56.8 | (12.1) | 35.1 | 64.9 | 2.1 | (0.6) |  |  | 30.0 | (15.0) | 76.6 |  |  |  |
|  | Minimal symptoms | 152 | 59.0 | (12.3) | 32.9 | 67.1 | 2.0 | (0.6) |  |  | 29.2 | (14.7) |  |  |  |  |
|  | Mild symptoms | 76 | 55.2 | (11.9) | 36.8 | 63.2 | 2.1 | (0.6) |  |  | 31.5 | (15.8) |  |  |  |  |
|  | Depression | 80 | 54.1 | (11.0)** | 37.5 | 62.5 | 2.4 | (0.5)*** |  |  | 29.8 | (15.0) |  |  |  |  |
| Carson et al. 2009 [28] | Total cohort | 1050 | 56.8 | (13.0) | 40.0 | 60.0 |  |  | 0.0 | 100 | 24.2 | (6.9) |  |  |  |  |
| Chan et al. 2010 [29] | Total cohort | 324 | 67.9 | (11.1) | 29.0 | 71.0 |  |  | 0.0 | 100 | 29.4 | (10.0) | 100.0 | 0.0 | 0.0 |  |
| Comín-Colet at al. 2016 [30] | Total cohort | 1037 | 70.6 | (11.1) | 30.1 | 69.9 |  |  | 54.9 | 45.1 | 33.7 | (6.8) | 100.0 | 0.0 | 0.0 |  |
|  | Better HRQoL | 696 | 69.2 | (11.2) | 25.3 | 74.7 |  |  | 71.8 | 28.2 | 34.4 | (6.4) | 100.0 | 0.0 | 0.0 |  |
|  | Worse HRQoL | 327 | 73.6 | (10.2)*** | 39.9*** | 60.1 |  |  | 18.5*** | 81.5*** | 32.2 | (7.5)*** | 100.0 | 0.0 | 0.0 |  |
| Comín-Colet et al. 2013 [14] | Total cohort | 552 | 72.0 | (11.0) | 43.0 | 57.0 |  |  | 61.0 | 40.0 | 45.0 | (16.0) |  |  |  | *b* |
|  | With ID | 349 | 73.0 | (10.0) | 46.0 | 54.0 |  |  | 54.0 | 46.0 | 47.0 | (17.0) |  |  |  |  |
|  | Without ID | 203 | 70.0 | (12.0) ** | 38.0 | 62.0 |  |  | 73.0 | 27.0 | 43.0 | (15.0)** |  |  |  |  |
| Cully et al. 2010 [31] | Total cohort | 96 | 71.9 | (7.83) | 1.0 | 99.0 | 3.4 | (0.68) | 11.5 | 88.6 |  |  |  |  |  |  |
|  | S depression | 48 | 70.9 | (7.66) |  |  | 3.4 | (0.62) | 6.2 | 93.8 |  |  |  |  |  |  |
|  | NS depression | 48 | 72.9 | (7.96) |  |  | 3.3 | (0.74) | 16.7 | 83.4 |  |  |  |  |  |  |
| Enjuanes et al. 2014 [32] | Total cohort | 1278 | 68.0 | (12.0) | 31.0 | 69.0 |  |  | 50.0 | 50.0 | 38.0 | (15.0) |  |  |  | *b* |
|  | With ID | 741 | 70.0 | (11.0) | 38.0 | 62.0 |  |  | 42.0 | 57.0 | 39.0 | (16.0) |  |  |  |  |
|  | Without ID | 537 | 66.0 | (13.0)*** | 22 | 78.0*** |  |  | 60.0 | 39.0 | 37.0 | (14.0) |  |  |  |  |
| Fotos et al. 2013 [16] | Total cohort | 199 |  |  | 37.7 | 62.3 |  |  | 0.0 | 100.0 |  |  |  |  |  |  |
| Fritschi & Redeker 2015 [33] | Total cohort | 173 | 60.4 | (16.1) | 34.7 | 65.3 |  |  |  |  | 32.6 | (15.2) |  |  |  |  |
|  | With DM | 54 | 62.9 | (14.3) | 27.8 | 72.2 |  |  | 44.4 | 55.6 | 33.5 | (15.5) |  |  |  |  |
|  | Without DM | 119 | 59.2 | (16.7) | 37.8 | 62.2 |  |  | 63.9 | 36.1 | 32.2 | (15.1) |  |  |  |  |
| Gastelurrutia et al. 2013 [34] | Total cohort | 1405 | 66.7 | (12.4) | 27.8 | 72.2 |  |  | 68.0 | 32.0 | 33.0 | (13.0) |  |  |  |  |
| Harrow et al. 2011 [35] | Total cohort | 1962 |  |  |  |  |  |  |  |  |  |  |  |  |  |  |
| Iqbal et al. 2010 [36] | Total cohort | 179 | 71.0 | (1.0) | 28 | 72.0 |  |  | 59.0 | 41.0 |  |  |  |  |  |  |
|  | Good HRQoL | 85 | 73.0 | (1.0) | 36 | 64.0 |  |  | 78.0 | 22.0 |  |  |  |  |  |  |
|  | Poor HRQoL | 94 | 69.0 | (2.0)* | 19 | 81.0* |  |  | 39.0*** | 61.0*** |  |  |  |  |  |  |
| Moliner et al. 2017 [37] | IIS | 219 | 66.0 | (12.0) | 35 | 65.0 |  |  | 56.0 | 44.0 | 33.0 | (11.0) |  | 12.0 |  |  |
|  | IIT | 454 | 68.0 | (12.0) | 31 | 69.0 |  |  | 42.0 | 58.0 | 37.0 | (16.0) |  | 28.0 |  |  |
|  | IIS+IIT | 389 | 70.0 | (13.0) | 39 | 61.0 |  |  | 36.0 | 64.0 | 38.0 | (17.0) |  | 32.0 |  |  |
|  | NIS | 759 | 62.0 | (13.0)*** | 21 | 79.0*** |  |  | 58.0 | 42.0*** | 33.0 | (13.0)*** |  | 18.0*** |  |  |
| Pantilant et al. 2016 [15] | Total cohort | 104 | 53.0 | (14.2) | 34 | 66.0 |  |  | 57.0 | 43.0 |  |  |  | 29.0 |  |  |
| Smolderen et al. 2009 [38] | Total cohort | 188 | 66.9 | (8.9) | 21.8 | 78.2 |  |  | 89.4 | 10.6 | 33.7 | (7.3) | 100.0 | 0.0 | 0.0 |  |
| Staniute et al. 2015 [39] | Total cohort | 855 | 58.6 | (8.8) | 26 | 74.0 |  |  | 83.2 | 16.8 |  |  | 14.0 |  |  |  |
| Streng et al. 2018 [8] | Total cohort | 3499 | 71.0 | (12.0) | 29.7 | 70.3 |  |  | 49.3 | 43.6 |  |  | 65.99 | 18.12 | 15.89 | *b* |
|  | Reduced EF | 2309 | 69.0 | (12.2) | 24.5 | 75.5 |  |  | 52.3 | 38.9 |  |  | 100.0 | 0.0 | 0.0 |  |
|  | Mid-range EF | 634 | 75.0 | (11.1) | 34.4 | 65.6 |  |  | 48.1 | 47.3 |  |  | 0.0 | 100.0 | 0.0 |  |
|  | Preserved EF | 556 | 78.0 | (9.8)*** | 46.0 | 54.0*** |  |  | 37.8 | 58.3 |  |  | 0.0 | 0.0 | 100.0 |  |
| Wienbergen et al. 2018 [40] | With ID | 516 | 70.1 | (12.0) | 30.9 | 69.1 |  |  | 26.4 | 73.6 |  |  | 100.0 | 0.0.0 | 0.0 |  |
|  | Without ID | 433 | 68.8 | (12.5) | 17.9*** | 82.1 |  |  | 33.2 | 66.8 |  |  | 100.0 | 0.0.0 | 0.0 |  |

COPD (chronic obstructive pulmonary disease), DM (diabetes mellitus), EF (ejection fraction), F (female), HRQoL (health-related quality of life), ID (iron deficiency), IIS (impaired iron storage), IIT (impaired iron transport), LVEF (left ventricular ejection fraction), M (male), NIS (normal iron status), NYHA (New York Heart Association). Missing information accounts for a lack of data availability within selected studies.

**^a^** Age group <40, 40–49, 50–59, 60–69 and more than 70 years old (*P* = 0.041), **^b^** NYHA class I, II, III and IV (*P* < 0.001)

Significant differences between groups: **P* < 0.05, ***P* < 0.01, ****P* < 0.001

**Table 3.** Etiology of heart failure in studies

|  | **Groups** | **IHD (%)** | **HHD (%)** | **VHD (%)** | **DCM (%)** | **ACM (%)** | **DrCM (%)** | **Idiopathic (%)** |
| --- | --- | --- | --- | --- | --- | --- | --- | --- |
| Bektas et al., 2017 [18] | Total cohort | 49.0 | 18.0 |  | 19.0 |  |  |  |
|  | With COPD | 43.0 | 20.0 |  | 21.0 |  |  |  |
|  | Without COPD | 59.0 | 15.0 |  | 15.0 |  |  |  |
| Bhatt et al. 2016 [27] | Total cohort | 40.3 |  |  |  |  |  |  |
|  | Minimal symptoms | 40.1 |  |  |  |  |  |  |
|  | Mild symptoms | 46.1 |  |  |  |  |  |  |
|  | Depression | 35.0 |  |  |  |  |  |  |
| Carson et al. 2009 [28] | Total cohort | 23.0 | 38.7 |  |  |  |  | 26.1 |
| Comín-Colet at al. 2016 [30] | Total cohort | 50.8 |  |  |  |  |  |  |
|  | Better HRQoL | 49.6 |  |  |  |  |  |  |
|  | Worse HRQoL | 53.5 |  |  |  |  |  |  |
| Comín-Colet et al. 2013 [14] | Total cohort | 40.0 |  |  |  |  |  |  |
|  | With ID | 41.0 |  |  |  |  |  |  |
|  | Without ID | 37.0 |  |  |  |  |  |  |
| Enjuanes et al. 2014 [32] | Total cohort | 53.0 |  |  |  |  |  |  |
|  | With ID | 55.0 |  |  |  |  |  |  |
|  | Without ID | 50.0 |  |  |  |  |  |  |
| Gastelurrutia et al. 2013 [34] | Total cohort | 52.8 | 9.5 | 9.4 | 12.2 | 5.3 | 2.5 |  |
| Iqbal et al. 2010 [36] | Total cohort | 62.0 |  |  |  |  |  |  |
|  | Good HRQoL | 62.0 |  |  |  |  |  |  |
|  | Poor HRQoL | 61.0 |  |  |  |  |  |  |
| Moliner et al. 2017 [37] | IIS | 65.0 |  |  |  |  |  |  |
|  | IIT | 53.0 |  |  |  |  |  |  |
|  | IIS + IIT | 55.0 |  |  |  |  |  |  |
|  | NIS | 59.0* |  |  |  |  |  |  |
| Wienbergen et al. 2018 [40] | With ID | 57.4 |  | 7.9 |  |  |  |  |
|  | Without ID | 53.1 |  | 4.6* |  |  |  |  |

ACM (alcoholic cardiomyopathy), COPD (chronic obstructive pulmonary disease), DCM (dilated cardiomyopathy), DM (diabetes mellitus), DrCM (drug-related cardiomyopathy), EF (ejection fraction), HHD (hypertensive heart disease), HRQoL (health-related quality of life), ID (iron deficiency), IHD (ischemic heart disease), IIS (impaired iron storage), IIT (impaired iron transport), NIS (normal iron status), VHD (valvular heart disease). Missing information accounts for a lack of data availability within selected studies.

Significant differences between groups: **P* < 0.05, ***P <* 0.01, ****P* < 0.001**Table 4.** Comorbidities in the studies

|  | **Groups** | **IHD (%)** | **VHD (%)** | **PVD (%)** | **AF (%)** | **Cancer (%)** | **PsyD (%)** | **Depression (%)** | **Anxiety (%)** | **CVA (%)** | **DM (%)** | **MSK (%)** | **HCh (%)** | **HT (%)** | **Anemia (%)** | **ID (%)** | **Kidney D (%)** | **Lung D (%)** | **Obesity (%)** | **Thyroid D (%)** | **Stomach D (%)** | **Liver D (%)** |
| --- | --- | --- | --- | --- | --- | --- | --- | --- | --- | --- | --- | --- | --- | --- | --- | --- | --- | --- | --- | --- | --- | --- |
| Ancheta et al. 2009 [17] | Total cohort |  |  |  |  |  |  | 61.0 |  |  |  |  |  |  |  |  | 0.0 |  |  |  |  |  |
| Arnold et al. 2016 [26] | Poor outcome |  | 55.9 |  |  | 13.5 |  |  |  |  | 5.4 |  |  |  |  |  |  |  |  |  |  |  |
|  | Acceptable outcome |  | 59.8 |  |  | 12.4* |  |  |  |  | 3.6* |  |  |  |  |  |  |  |  |  |  |  |
| Bektas et al., 2017 [18] | Total cohort |  |  | 3.0 |  |  |  |  |  | 9.0 | 26.0 |  | 72.0 | 61.0 |  |  |  | 63.4 |  |  |  |  |
|  | With COPD |  |  | 3.0 |  |  |  |  |  | 9.0 | 27.0 |  | 73.0 | 65.0 |  |  |  | 0.0 |  |  |  |  |
|  | Without COPD |  |  | 4.0 |  |  |  |  |  | 9.0 | 25.0 |  | 69.0 | 54.0 |  |  |  | 100.0 |  |  |  |  |
| Bhatt et al. 2016 [27] | Total cohort |  |  |  |  |  |  | 26.0 |  |  | 33.4 |  |  |  |  |  |  |  |  |  |  |  |
|  | Minimal symptoms |  |  |  |  |  |  | 0.0 |  |  | 30.9 |  |  |  |  |  |  |  |  |  |  |  |
|  | Mild symptoms |  |  |  |  |  |  | 0.0 |  |  | 30.3 |  |  |  |  |  |  |  |  |  |  |  |
|  | Depression |  |  |  |  |  |  | 100.0 |  |  | 41.2 |  |  |  |  |  |  |  |  |  |  |  |
| Carson et al. 2009 [28] | Total cohort |  |  |  |  |  |  |  |  |  | 40.9 |  |  |  |  |  |  |  |  |  |  |  |
| Chan et al. 2010 [29] | Total cohort | 50.0 |  |  | 20.4 |  |  |  |  |  | 32.1 |  |  |  |  |  |  |  |  |  |  |  |
| Comín-Colet at al. 2016 [30] | Total cohort |  |  |  | 45.5 |  |  |  |  |  | 44.0 |  |  | 79.2 | 21.3 |  | 23.5 |  |  |  |  |  |
|  | Better HRQoL |  |  |  | 42.1 |  |  |  |  |  | 41.4 |  |  | 77.4 | 17.1 |  | 18.1 |  |  |  |  |  |
|  | Worse HRQoL |  |  |  | 52.8** |  |  |  |  |  | 48.9* |  |  | 82.9* | 30.6*** |  | 35.2*** |  |  |  |  |  |
| Comín-Colet et al. 2013 [14] | Total cohort |  |  |  |  |  |  |  |  |  | 43.0 |  |  | 78.0 | 37.0 | 63.2 | 55.0 |  |  |  |  |  |
|  | With ID |  |  |  |  |  |  |  |  |  | 47.0 |  |  | 80.0 | 44.0 | 100.0 | 56.0 |  |  |  |  |  |
|  | Without ID |  |  |  |  |  |  |  |  |  | 37.0* |  |  | 74.0 | 26*** | 0.0 | 52.0 |  |  |  |  |  |
| Cully et al. 2010 [31] | Total cohort |  |  |  |  |  |  | 50.0 |  |  |  |  |  |  |  |  |  |  |  |  |  |  |
|  | S depression |  |  |  |  |  |  | 100.0 |  |  |  |  |  |  |  |  |  |  |  |  |  |  |
|  | NS depression |  |  |  |  |  |  | 0.0 |  |  |  |  |  |  |  |  |  |  |  |  |  |  |
| Enjuanes et al. 2014 [32] | Total cohort |  |  |  |  |  |  |  |  |  | 38.0 |  |  | 59.0 | 35.0 | 58.0 | 29.0 |  |  |  |  |  |
|  | With ID |  |  |  |  |  |  |  |  |  | 42.0 |  |  | 60.0 | 42.0 | 100.0 | 29.0 |  |  |  |  |  |
|  | Without ID |  |  |  |  |  |  |  |  |  | 34.0** |  |  | 58.0 | 26.0*** | 0 | 26.0** |  |  |  |  |  |
| Fotos et al. 2013 [16] | Total cohort |  |  |  |  | 7.0 | 8.0 |  |  |  | 47.7 |  |  | 76.4 |  |  | 14.1 | 26.1 |  |  |  |  |
| Fritschi & Redeker 2015 [33] | Total cohort |  |  |  |  |  |  |  |  |  | 31.2 |  |  |  |  |  |  |  |  |  |  |  |
|  | With DM |  |  |  |  |  |  |  |  |  | 100.0 |  |  |  |  |  |  |  |  |  |  |  |
|  | Without DM |  |  |  |  |  |  |  |  |  | 0.0 |  |  |  |  |  |  |  |  |  |  |  |
| Gastelurrutia et al. 2013 [34] | Total cohort |  |  | 16.2 | 18.0 |  |  | 31.2 |  |  | 39.2 |  |  | 60.9 | 32.4 |  | 54.5 | 18.3 |  |  |  |  |
| Iqbal et al. 2010 [36] | Total cohort |  |  |  |  | 8.0 |  | 5.1 |  | 6.8 | 29.0 | 9.6 |  | 44.0 |  |  |  | 8.5 |  |  |  |  |
|  | Good HRQoL |  |  |  |  | 10.0 |  | 4.4 |  | 6.7 | 24.0 | 6.6 |  | 44.0 |  |  |  | 4.4 |  |  |  |  |
|  | Poor HRQoL |  |  |  |  | 6.0 |  | 5.7 |  | 6.9 | 34.0 | 12.6 |  | 44.0 |  |  |  | 12.6 |  |  |  |  |
| Moliner et al. 2017 [37] | IIS |  |  |  | 31.0 |  |  |  |  |  | 29.0 |  |  | 54.0 | 20.0 | 100.0 | 20.0 |  |  |  |  |  |
|  | IIT |  |  |  | 32.0 |  |  |  |  |  | 44.0 |  |  | 61.0 | 43.0 | 100.0 | 36.0 |  |  |  |  |  |
|  | IIS + IIT |  |  |  | 34.0 |  |  |  |  |  | 42.0 |  |  | 63.0 | 48.0 | 100.0 | 33.0 |  |  |  |  |  |
|  | NIS |  |  |  | 34.0 |  |  |  |  |  | 32.0*** |  |  | 57.0 | 21.0*** | 0.0 | 22.0*** |  |  |  |  |  |
| Pantilant et al. 2016 [15] | Total cohort |  |  |  |  | 8.0 |  | 45.0 |  |  | 23.0 | 15.0 |  | 36.0 | 15.0 |  | 15.0 | 16.0 |  |  | 8.0 | 5.0 |
| Smolderen et al. 2009 [38] | Total cohort | 62.4 |  | 9.0 |  |  |  |  |  | 14.5 | 29.0 |  | 64.0 | 43.0 |  |  | 3.2 | 20.4 |  |  |  |  |
| Staniute et al. 2015 [39] | Total cohort | 59.2 |  |  |  |  |  | 13.1 | 32.9 |  |  |  |  | 80.0 |  |  |  |  |  |  |  |  |
| Streng et al. 2018 [8] | Total cohort | 42.4 |  | 16.7 | 45.3 |  |  |  |  | 13.0 | 32.6 |  |  | 60.9 | 36.2 |  | 50.3 | 17.7 | 32.8 | 12.5 |  |  |
|  | Reduced EF | 43.2 |  | 14.0 | 43.2 |  |  |  |  | 11.1 | 31.3 |  |  | 56.5 | 32.8 |  | 48.3 | 16.6 | 29.4 | 10.9 |  |  |
|  | Mid-range EF | 47.8 |  | 20.0 | 49.5 |  |  |  |  | 16.9 | 34.9 |  |  | 70.0 | 40.1 |  | 52.7 | 16.2 | 36.8 | 13.7 |  |  |
|  | Preserved EF | 32.6* |  | 24.3*** | 49.6*** |  |  |  |  | 16.4*** | 35.6 |  |  | 69.4*** | 45.5*** |  | 56.1** | 23.7*** | 42.3*** | 17.4*** |  |  |
| Wienbergen et al. 2018 [40] | With ID | 57.4 | 7.9 |  | 40.5 | 11.0 |  |  |  |  | 37.6 |  |  |  | 48.5 | 100 | 40.5 | 16.7 |  |  |  |  |
|  | Without ID | 53.1 | 4.6* |  | 40.6 | 10.9 |  |  |  |  | 35.1 |  |  |  | 37.6 | 0.0 | 40.6 | 16.4 |  |  |  |  |

AF (atrial fibrillation), COPD (chronic obstructive pulmonary disease), CVA (cerebrovascular accident), D (disease), DM (diabetes mellitus), EF (ejection fraction), HCh (hypercholesterolemia), HRQoL (health-related quality of life), HT (hypertension), ID (iron deficiency), IHD (ischemic heart disease), IIS (impaired iron storage), IIT (impaired iron transport), MSK (musculoskeletal), NIS (normal iron status), PsyD (psychiatric disease), PVD (peripheral vascular disease), S (symptomatic), NS (non-symptomatic), VHD (valvular heart disease). Missing information accounts for a lack of data availability within selected studies.

Significant differences between groups: **P* < 0.05, ***P* < 0.01, ****P* < 0.001

**Table 5.** Pharmacological treatment in the studies

|  | **Groups** | **Β-blocker (%)** | **Ivabradine (%)** | **Nitrates (%)** | **ACEI (%)** | **ARB (%)** | **ACEI/ARB (%)** | **Statins (%)** | **CCB (%)** | **C03-Diuretics (%)** | **C03DA-AA (%)** | **CC01AA-Digitalis (%)** | **C01B-Antiarrythmic (%)** | **B01A-Antythrombotic (%)** | **B01AC-Antiplatelet (%)** | **Antianginal agents (%)** | **N06A-Antidepressants (%)** | **Anxiety medication (%)** | **Diabetes medication (%)** | **Pain medication (%)** | **Iron medication (%)** | **Inhalation therapy (%)** |
| --- | --- | --- | --- | --- | --- | --- | --- | --- | --- | --- | --- | --- | --- | --- | --- | --- | --- | --- | --- | --- | --- | --- |
| Bektas et al., 2017 [18] | Total cohort | 93.0 |  |  | 59.0 | 32.0 |  |  |  | 79.0 | 40.0 |  |  |  |  |  |  |  |  |  |  | 18.0 |
|  | With COPD | 92.0 |  |  | 59.0 | 30.0 |  |  |  | 77.0 | 36.0 |  |  |  |  |  |  |  |  |  |  | 9.0 |
|  | Without COPD | 93.0 |  |  | 60.0 | 35.0 |  |  |  | 82.0 | 47.0 |  |  |  |  |  |  |  |  |  |  | 35.0*** |
| Bhatt et al. 2016 [27] | Total cohort | 93.5 |  |  |  |  | 78.9 |  |  | 84.3 | 43.2 |  |  |  |  |  | 25.6 |  |  |  |  |  |
|  | Minimal symptoms | 93.4 |  |  |  |  | 76.3 |  |  | 80.0 | 40.1 |  |  |  |  |  | 15.1 |  |  |  |  |  |
|  | Mild symptoms | 94.7 |  |  |  |  | 82.9 |  |  | 84.0 | 48.7 |  |  |  |  |  | 25.0 |  |  |  |  |  |
|  | Depression | 92.5 |  |  |  |  | 80 |  |  | 92.5* | 43.8 |  |  |  |  |  | 46.3*** |  |  |  |  |  |
| Carson et al. 2009 [28] | Total cohort | 87.0 |  |  | 78.0 | 28.0 | 93 |  |  | 94.0 | 39.0 | 62.0 |  |  |  |  |  |  |  |  |  |  |
| Chan et al. 2010 [29] | Total cohort | 86.1 |  |  |  |  | 91.7 | 57.4 |  | 82.7 | 37.0 |  | 19.8 |  |  |  |  |  |  |  |  |  |
| Comín-Colet at al. 2016 [30] | Total cohort | 76.6 | 8.8 |  |  |  | 89.6 | 75.8 |  | 89.2 | 66.4 | 21.7 |  | 39.9 | 60.0 |  |  |  |  |  |  |  |
|  | Better HRQoL | 78.2 | 9.2 |  |  |  | 91.0 | 76.6 |  | 86.9 | 64.8 | 19.7 |  | 36.4 | 60.2 |  |  |  |  |  |  |  |
|  | Worse HRQoL | 72.8 | 8.3 |  |  |  | 86.5* | 73.4 |  | 93.6** | 69.7 | 26.0* |  | 47.7** | 59.0 |  |  |  |  |  |  |  |
| Comín-Colet et al. 2013 [14] | Total cohort | 92.0 |  |  |  |  | 80.0 |  |  | 87.0 | 36.0 | 11.0 |  |  |  |  |  |  |  |  |  |  |
|  | With ID | 91.0 |  |  |  |  | 78.0 |  |  | 86.0 | 33.0 | 9.0 |  |  |  |  |  |  |  |  |  |  |
|  | Without ID | 93.0 |  |  |  |  | 83.0 |  |  | 89.0 | 40.0 | 13.0 |  |  |  |  |  |  |  |  |  |  |
| Enjuanes et al. 2014 [32] | Total cohort | 88.0 |  |  |  |  | 86.0 | 61.0 |  | 79.0 | 32.0 | 17.0 |  | 43.0 | 48.0 |  |  |  |  |  |  |  |
|  | With ID | 87.0 |  |  |  |  | 85.0 | 62.0 |  | 83.0 | 39.0 | 17.0 |  | 48.0 | 48.0 |  |  |  |  |  |  |  |
|  | Without ID | 89.0 |  |  |  |  | 90** | 61.0 |  | 74*** | 37.0 | 19.0 |  | 44.0 | 48.0 |  |  |  |  |  |  |  |
| Fotos et al. 2013 [16] | Total cohort | 70.3 |  |  | 73.4 | 41.7 |  |  | 42.7 | 88.9 |  | 30.6 |  |  |  |  |  |  |  |  |  |  |
| Gastelurrutia et al. 2013] [34] | Total cohort | 67.8 |  |  |  |  | 75.2 |  |  | 76.4 |  |  |  |  |  |  |  |  |  |  |  |  |
| Iqbal et al. 2010 [36] | Total cohort | 56.0 |  |  | 71.0 |  |  | 55.0 |  | 93.0 | 33.0 | 46.0 |  |  |  |  |  |  |  |  |  |  |
|  | Good HRQoL | 66.0 |  |  | 72.0 |  |  | 51.0 |  | 94.0 | 12.0 | 42.0 |  |  |  |  |  |  |  |  |  |  |
|  | Poor HRQoL | 45.0** |  |  | 70.0 |  |  | 59.0 |  | 92.0 | 21* | 51.0 |  |  |  |  |  |  |  |  |  |  |
| Moliner et al. 2017 [37] | IIS | 93.0 |  |  |  |  | 91.0 | 70.0 |  | 70.0 | 54.0 | 23.0 |  |  | 82.0 |  |  |  |  |  |  |  |
|  | IIT | 87.0 |  |  |  |  | 85.0 | 61.0 |  | 87.0 | 48.0 | 23.0 |  |  | 83.0 |  |  |  |  |  |  |  |
|  | IIS + IIT | 88.0 |  |  |  |  | 85.0 | 60.0 |  | 87.0 | 42.0 | 17.0 |  |  | 89.0 |  |  |  |  |  |  |  |
|  | NIS | 93.0*** |  |  |  |  | 93.0*** | 67.0* |  | 75.0*** | 56*** | 30.0*** |  |  | 85.0 |  |  |  |  |  |  |  |
| Pantilant et al. 2016 [15] | Total cohort | 18.4 |  |  |  |  | 80.6 | 55.3 |  | 78.6 | 44.7 |  |  |  |  | 25.2 | 18.4 | 9.7 | 19.4 | 17.5 |  |  |
| Smolderen et al. 2009 [38] | Total cohort | 66.8 |  | 36.9 | 59.9 |  |  | 68.4 | 16.0 | 64.7 |  |  |  | 62.6 | 32.6 |  |  |  |  |  |  |  |
| Wienbergen et al. 2018 [40] | With ID | 86.0 |  |  |  |  | 84.0 |  |  | 85.5 | 53.9 |  |  |  |  |  |  |  |  |  | 9.3 |  |
|  | Without ID | 92.5* |  |  |  |  | 87.0 |  |  | 75.5** | 61.7 |  |  |  |  |  |  |  |  |  | 2.8** |  |

ACEI (angiotensin converting enzyme inhibitors), ARB (angiotensin II receptor blockers), COPD (chronic obstructive pulmonary disease), DM (diabetes mellitus), EF (ejection fraction), HRQoL (health-related quality of life), ID (iron deficiency), IIS (impaired iron storage), IIT (impaired iron transport), NIS (normal iron status). Missing information accounts for a lack of data availability within studies.

Significant differences between groups: **P* < 0.05, ***P* < 0.01, ****P* < 0.001

**Table 6.** Cardiac devices and interventions in studies

|  | **Groups** | **ICD (%)** | **ICD and/or CRT (%)** | **Previous cardiac surgery (%)** | **CABG (%)** | **PCI/PTCA (%)** |
| --- | --- | --- | --- | --- | --- | --- |
| Arnold et al. 2016 [26] | Poor outcome |  |  | 47.8 |  |  |
|  | Acceptable outcome |  |  | 39.8** |  |  |
| Bhatt et al. 2016 [27] | Total cohort |  | 65.3 |  |  |  |
|  | Minimal symptoms |  | 63.2 |  |  |  |
|  | Mild symptoms |  | 65.8 |  |  |  |
|  | Depression |  | 68.8 |  |  |  |
| Chan et al. 2010 [29] | Total cohort | 9.6 |  |  | 29.9 | 24.7 |
| Fotos et al. 2013 [16] | Total cohort |  |  | 6.5 |  |  |
| Smolderen et al. 2009 [38] | Total cohort |  |  |  | 28.0 | 24.2 |
| Streng et al. 2018 [8] | Total cohort |  |  |  | 17.3 | 19.7 |
|  | Reduced EF |  |  |  | 17.2 | 20.4 |
|  | Mid-range EF |  |  |  | 20.3 | 20.2 |
|  | Preserved EF |  |  |  | 13.9* | 16* |

CABG (coronary artery bypass graft), COPD (chronic obstructive pulmonary disease), CRT (cardiac resynchronization therapy), DM (diabetes mellitus), EF (ejection fraction), ICD (implantable cardioverter-defibrillator), ID (iron deficiency), IIS (impaired iron storage), IIT (impaired iron transport), NIS (normal iron status), PCI (percutaneous coronary intervention), PTCA (percutaneous transluminal coronary angioplasty). Missing information accounts for a lack of data availability within selected studies.

Significant differences between groups: **P* < 0.05, ***P* < 0.01, ****P* < 0.001

**Table 7.** Anemia and iron deficiency and quality of life

|  | **Comorbidity** | **Definition** | **Quality of life**  **assessment** | **Without comorbidity** | | **With comorbidity** | | **Δ (%)** | **Statistical test** | ***P*** | **Interpretation** |
| --- | --- | --- | --- | --- | --- | --- | --- | --- | --- | --- | --- |
| Comín-Colet at al. 2016 [30] | Anemia | Report | KCCQ SS <50 (worse) |  |  |  |  |  | Univariable binary logistic regression | ≤.001 | Worse HRQoL |
|  |  |  | EQ-5D Index <0.5 (worse) |  |  |  |  |  | Univariable binary logistic regression | ≤.001 | Worse HRQoL |
|  |  |  | EQ-5D VASS <50 (worse) |  |  |  |  |  | Univariable binary logistic regression | ≤.001 | Worse HRQoL |
| Comín-Colet et al. 2013 [14] | ID | Ferritin (ng/mL):  <100 or <800 + TSAT <20% | MLHFQ SS | 34.4 | (26.4) | 41.0 | (24.7) | 19.2 | Mann-Whitney U-test | ≤.01 | Worse HRQoL |
|  |  |  | MLHFQ SS ≥ median [42] |  |  |  |  |  | Univariable binary logistic regression | ≤.05 | Worse HRQoL |
|  |  |  | MLHFQ SS |  |  |  |  |  | Multivariate regression ^(i)^ | ≤.01 | Worse HRQoL |
|  |  |  | MLHFQ PS | 20.0 | (14.3) | 23.3 | (13.5) | 16.5 | Mann-Whitney U-test | ≤.001 | Worse HRQoL |
|  |  |  | MLHFQ PS ≥ median [42] |  |  | OR: 1.7; 95% CI: 1.2-2.4 | | | Univariable binary logistic regression | ≤.01 | Worse HRQoL |
|  |  |  | MLHFQ PS |  |  |  |  |  | Multivariate regression ^(i)^ | ≤.01 | Worse HRQoL |
|  |  |  | MLHFQ ES | 6.0 | (6.1) | 6.7 | (5.8) | 11.7 | Mann-Whitney U-test | ns | ns |
|  | Anemia | Hemoglobin (g/dL): ≤12 | MLHFQ SS | 35.2 | (26.3) | 44.2 | (23.1) | 25.6 | Mann-Whitney U-test | ≤.001 | Worse HRQoL |
|  |  |  | MLHFQ SS ≥ median [42] |  |  |  |  |  | Univariable binary logistic regression | ≤.001 | Worse HRQoL |
|  |  |  | MLHFQ SS |  |  |  |  |  | Multivariate regression ^(i)^ | ns | ns |
|  |  |  | MLHFQ PS ≥ median [42] |  |  | OR: 1.8; 95% CI: 1.3-2.6 | | | Univariable binary logistic regression | ≤.001 | Worse HRQoL |
|  |  |  | MLHFQ PS |  |  |  |  |  | Multivariate regression ^(i)^ | ns | ns |
|  |  |  | MLHFQ ES |  |  |  |  |  | Multivariate regression ^(i)^ | ns | ns |
| Enjuanes et al. 2014 [32] | ID | Ferritin (µg/mL):  <100 or 100-–299 + TSAT <20% | MLHFQ SS | 37.0 | (25.0) | 42.0 | (25.0) | 13.5 | Student's t-test/Mann-Whitney U-test | ≤.001 | Worse HRQoL |
|  |  |  | MLHFQ SS |  |  |  |  |  | Univariate linear regression | ≤.001 | Worse HRQoL |
|  |  |  | MLHFQ SS |  |  |  |  |  | Multivariable linear regression ^(ii)^ | ≤.01 | Worse HRQoL |
|  |  | + no anemia | MLHFQ SS ≥ 55 (worse) |  |  | OR: 1.6; 95% CI: 1.1-2.6^€^ | | | Multivariate binary logistic regression ^(ii)^ | ≤.05 | Worse HRQoL |
|  | Anemia | Hemoglobin (g/dL):  <12 (F) and <13 (M) | MLHFQ SS |  |  |  |  |  | Univariate linear regression | ≤.001 | Worse HRQoL |
|  |  |  | MLHFQ SS |  |  |  |  |  | Multivariable linear regression ^(ii)^ | ns | ns |
|  |  | + no ID | MLHFQ SS ≥ 55 (worse) |  |  | OR: 1.1; 95% CI: 0.6-2.1^€^ | | | Multivariate binary logistic regression ^(ii)^ | ns | ns |
| Harrow et al. 2011 [35] | Anemia | Baseline hemoglobin (mg/dL):  <12 vs >14 [ref] | SF-6D HU 36m change |  |  |  |  |  | Multiple linear regression ^(iii)^ | ns | ns |
| Moliner et al. 2017 [37] | IIS + IIT | Ferritin <100µg/mL + TSAT <20% | MLHFQ SS >median [55] (worse) |  |  | OR: 1.8; 95% CI: 1.2-2.7^$^ | | | Multivariable logistic regression ^(iv)^ | ≤.01 | Worse HRQoL |
|  | Isolated IIT | Ferritin ≥100µg/mL + TSAT <20% | MLHFQ SS >median [55] (worse) |  |  | OR: 1.7; 95% CI: 1.2-2.5^$^ | | | Multivariable logistic regression ^(iv)^ | ≤.01 | Worse HRQoL |
|  | Isolated IIS | Ferritin <100µg/mL + TSAT ≥20% | MLHFQ SS >median [55] (worse) |  |  | OR: 1.1; 95% CI: 0.6-1.8^$^ | | | Multivariable logistic regression ^(iv)^ | ns | ns |
| Streng et al. 2018 [8] | Anemia | Hemoglobin (g/dL):  <12 (F) and <13 (M) | KCCQ SS | 47.0 | [31–65] | 41.0 | [27–56] | -12.8 | Univariable linear regression | ≤.001 | Worse HRQoL |
|  |  |  | KCCQ SS |  |  |  |  |  | Multivariable linear regression ^(v)^ | ≤.001 | Worse HRQoL |
|  |  |  | EQ-5D VASS | 60.0 | [45–70] | 50.0 | [40–70] | -16.7 | Univariable linear regression | ≤.001 | Worse HRQoL |
| Wienbergen et al. 2018 [40] | ID | Ferritin (µg/mL):  <100 or 100–299 + TSAT <20% | EQ-5D VASS at 12m | 70.0 | [50–80] | 60.0 | [50–75] | -14.3 | Pearson's Chi-square/Mann-Whitney test | ≤.05 | Worse HRQoL |
|  |  |  | EQ-5D VASS > 12m before (good) |  |  |  |  |  | Pearson's Chi-square/Mann-Whitney test | ≤.05 | Worse HRQoL ^(I)^ |
|  |  |  | EQ-5D VASS < 12m before (bad) |  |  |  |  |  | Pearson's Chi-square/Mann-Whitney test | ns | ns |

CI (confidence interval) ES (emotional score), F (female), HRQoL (health-related quality of life), HU (health utilities), ID (iron deficiency), IIS (impaired iron storage ), IIT (impaired iron transport), KCCQ (Kansas City Cardiomyopathy Questionnaire), M (male), MLHFQ (Minnesota Living with Heart Failure Questionnaire), ns (not significant), OR (odds ratio), PS (physical score), SS (summary score), TSAT (transferrin saturation), VASS (visual analogue scale score). Missing information accounts for a lack of data availability within selected studies.

**^$^** Reference is normal iron status, **^€^** Reference is absence of anemia and ID

**^(i)^** Including anemia and iron deficiency, and adjusted for age, gender, left ventricular ejection fraction (LVEF), systolic blood pressure (SBP), heart rate ≥70, CKD, hospital admission related to heart failure (HF) in the previous 6 months, ischemic chronic HF etiology, dependency [Barthel Index <90], hypertension, diabetes mellitus, angiotensin converting enzyme inhibitors (ACEI) or angiotensin II receptor blockers (ARB), loop diuretics, N-terminal pro b-type natriuretic peptide (NT-proBNP) ≥ median [1620 pg/mL], c-reactive protein (CRP) ≥median [0.8mg/dL], New York Heart Association (NYHA) class [II-III and IV], anemia [Hb ≤ 12g/dL], iron deficiency, and sTfR ≥median [1.62mg/L]; **^(ii)^** including iron deficiency and anemia adjusted for age, gender, LVEF, center [Poland vs Holland and Spain vs Holland], body mass index (BMI) < 18.5kg/m2, SBP, heart rate, NYHA class [II-III vs I and IV vs I], preserved [<45%] vs reduced LVEF, left ventricular end diastolic diameter (LVEDD) > 55mm, HF etiology (ischemic vs non ischemic), hypertension, diabetes mellitus, anemia, iron deficiency, ACEI or ARB, aldosterone antagonists, statins, loop diuretics, antiplatelets, anticoagulants, Hb, transferrin saturation (TSAT), red cell distribution width (RDW), mean corpuscular hemoglobin (MCH), RDW>15% + MCV≤100fl, NT-proBNP, estimated glomerular filtration rate (eGFR), and CRP; **^(iii)^** including Hb change group (loss vs no loss), age, race, education level, BMI category, diabetes with medication, disability, rheumatoid arthritis without medication, hospitalization in the past 2 years, exercise level (METS), depression score, stroke or heart attack, cancer diagnosis, nonsteroidal anti-inflammatory drug use, and baseline Hb group; **^(iv)^** adjusted for center, sex, age, SBP, diabetes, HF etiology, LVEF, LVEDD, eGFR, serum NT-proBNP, Hb, CRP, BMI, NYHA class, treatment with, ARB, beta-blockers, statins, and diuretics; **^(v)^** adjusted for age, sex, and NYHA

**^(I)^** Reduced prevalence of patients with good evolution

**Table 8.** Mental and behavioral disorders and quality of life

|  | **Comorbidity** | **Definition** | **Quality of life** | **Without**  **comorbidity** | | **With**  **comorbidity** | | **Δ (%)** | **Statistical test** | **p** | **Interpretation** |
| --- | --- | --- | --- | --- | --- | --- | --- | --- | --- | --- | --- |
| Ancheta et al. 2009 [17] | Depression | CES-D score >16 | MLHFQ SS | 27.0 | (22.0) | 70.0 | (25.0) | 159.3 | Student's t-test/Mann-Whitney U-test | ≤.001 | Worse HRQoL |
|  |  |  | MLHFQ SS |  |  |  |  |  | Multivariate regression ^(i)^ | ≤.001 | Worse HRQoL |
|  |  |  | MLHFQ SS |  |  |  |  |  | Multivariate regression ^(ii)^ | ≤.001 | Worse HRQoL |
|  |  |  | MLHFQ PS | 12.0 | (11.0) | 27.0 | (12.0) | 125.0 | Student's t-test/Mann-Whitney U-test | ≤.001 | Worse HRQoL |
|  |  |  | MLHFQ ES | 5.0 | (6.0) | 15.0 | (7.0) | 200.0 | Student's t-test/Mann-Whitney U-test | ≤.001 | Worse HRQoL |
| Bhatt et al. 2016 [27] | Depression | PHQ-9 symptoms: 04 (minimal), 5–9 (mild), 10–27 (depression) | KCCQ SS | 80.0 | (20.0) | 45.0 | (21.0) | -43.8 | Analysis of variance (p for trend) ^(iii)^ | ≤.001 | Worse HRQoL^(I)^ |
|  |  |  | KCCQ SS <45 (worse) |  |  |  |  |  | Logistic regression (p for trend) ^(iii)^ | ≤.001 | Worse HRQoL^(II)^ |
|  |  |  | KCCQ CS | 83.0 | (16.0) | 53.0 | (22.0) | -36.1 | Analysis of variance (p for trend) ^(iii)^ | ≤.001 | Worse HRQoL^(I)^ |
| Cully et al. 2010 [31] | Depression | GDS score ≥6 | KCCQ SS | 59.9 | (19.8) | 46.3 | (20.9) | -22.8 | Student's t-test | ≤.001 | Worse HRQoL |
|  |  |  | KCCQ PLS | 56.2 | (20.5) | 41.5 | (24.4) | -26.3 | Student's t-test | ≤.01 | Worse HRQoL |
|  |  |  | KCCQ SySS | 59.4 | (29.5) | 50.0 | (23.1) | -15.8 | Student's t-test | ns | ns |
|  |  |  | KCCQ SyFS | 55.7 | (25.3) | 46.1 | (22.8) | -17.3 | Student's t-test | ≤.05 | Worse HRQoL |
|  |  |  | KCCQ SyBS | 66.5 | (25.1) | 55.7 | (23.8) | -16.2 | Student's t-test | ≤.05 | Worse HRQoL |
|  |  |  | KCCQ SES | 76.6 | (25.9) | 77.3 | (23.6) | 1.0 | Student's t-test | ns | ns |
|  |  |  | KCCQ QoLS | 64.6 | (24.0) | 48.4 | (25.4) | -25.0 | Student's t-test | ≤.01 | Worse HRQoL |
|  |  |  | KCCQ SLS | 57.5 | (26.3) | 44.0 | (28.2) | -23.4 | Student's t-test | ≤.05 | Worse HRQoL |
| Fotos et al. 2013 [16] | Psychiatric disease | Report | MLHFQ SS | 62.0 | (20.3) | 71.8 | (19.0) | 15.8 | Student's t-test | ns | ns |
|  |  |  | MLHFQ SS |  |  |  |  |  | Multivariate linear regression ^(iv)^ | ≤.05 | Worse HRQoL |
| Gastelurrutia et al. 2013 [34] | Depression | Yesavage abbreviated GDS score ≥1 | MLHFQ SS | 27.6 | (17.9) | 39.6 | (18.2) | 43.5 | Student's t-test | ≤.001 | Worse HRQoL |
|  |  |  | MLHFQ SS |  |  |  |  |  | Multivariate linear regression ^(v)^ | ≤.001 | Worse HRQoL |
| Iqbal et al. 2010 [36] | Depression (%) ^(a)^ | Report | Good/poor HRQoL^(A)^ |  |  |  |  |  | Chi-square or Fisher's exact test | ns | ns |
| Pantilant et al. 2016 [15] | Depression | CES-D score ≥16 | Baseline MLHFQ SS | 36.7 | [31.1 - 42.3] | 65.3 | [59.6 - 71.0] | 77.9 | Analysis of variance | ≤.001 | Worse HRQoL |
|  |  |  | Baseline MLHFQ SS |  |  |  |  |  | Multivariate linear regression ^(vi)^ | ≤.001 | Worse HRQoL |
| Staniute et al. 2015 [39] | Depression symptoms | HADS-D score ≥8 | MLHFQ SS |  |  |  |  |  | Multivariate regression ^(vii)^ | ≤.001 | Worse HRQoL |
|  |  |  | MLHFQ SS |  |  |  |  |  | Mediation analysis Type D personality - HRQoL | ≤.001 | Worse HRQoL |
|  | Anxiety symptoms | HADS-A score ≥8 | MLHFQ SS |  |  |  |  |  | Multivariate regression ^(vii)^ | ≤.001 | Worse HRQoL |
|  |  |  | MLHFQ SS |  |  |  |  |  | Mediation analysis Type D personality - HRQoL | ≤.001 | Worse HRQoL |

CES-D (Center for Epidemiologic Studies Depression Scale), CS (clinical score), ES(emotional score), GDS (Geriatric Depression Scale), HADS-A (Hospital Anxiety and Depression Scale - Anxiety), HADS-D (Hospital Anxiety and Depression Scale - Depression), HRQoL (health-related quality of life), KCCQ (Kansas City Cardiomyopathy Questionnaire), MLHFQ (Minnesota Living with Heart Failure Questionnaire), PHQ-9 (Patient Health Questionnaire), PLS (physical limitation score), PS (physical score), QoLS (quality of life score), SES (self-efficacy score), SLS (social limitation score), SS (summary score), SyBS (symptom burden score), SyFS (symptom frequency score), SySS (symptom stability score). Missing information accounts for a lack of data availability within selected studies.

**^a^** Comorbidity prevalence as a function of outcome

**^A^** MLHFQ SS < median (good) or ≥ median (poor)

**^(i)^** including left ventricular ejection fraction (LVEF), plasma b-type natriuretic peptide (BNP), age, and depression score; **^(ii)^** including LVEF, plasma BNP, age, depression score, gender, New York Heart Association (NYHA) class, ethnicity, education, and marital status; **^(iii)^** adjusted for baseline age, gender, body mass index (BMI(, race, ejection fraction, heart failure (HF) etiology, diabetes mellitus, systolic blood pressure, sodium and creatinine levels, and HF treatment (devices and medications); **^(iv)^** including age, marital status, NYHA class [III or IV], diabetes mellitus, hypertension, chronic renal failure, chronic respiratory failure, cancer, psychiatric disease, cardiac interventions in the last 6 months, hospitalizations in the last year, and taking medication at the right time; **^(v)^** including abnormal Barthel index score [<90], abnormal Older American Resources and Services (OARS) scale score [<10F and <6M], abnormal Pfeiffer test score [>3±1], and depressive symptoms [GDS≥1]; **^(vi)^** including HF class, depression, dyspnea, and pain severity; **^(vii)^** adjusted for age, gender, acute myocardial infarction, NYHA class, anxiety symptoms, depressive symptoms, social support, and type D personality

**^I^** Worse quality of life with increasing depressive symptom severity; **^II^** greater prevalence of patients with poor HRQoL with increasing depressive symptom severity

**Table 9.** Diabetes mellitus and quality of life

|  | **Comorbidity** | **Definition** | **Quality of life**  **assessment** | **Without**  **comorbidity** | | **With comorbidity** | | **Δ (%)** | **Statistical test** | ***P*** | **Interpretation** |
| --- | --- | --- | --- | --- | --- | --- | --- | --- | --- | --- | --- |
| Arnold et al. 2016 [26] | Baseline severe diabetes (%) ^(a)^ | Report | Poor/acceptable outcome ^(A)^ |  |  |  |  |  | Chi-square test | ≤.05 | Worse HRQoL^(I)^ |
| Carson et al. 2009 [28] | Diabetes mellitus | Report | MLHFQ SS | 50.2 | (25.0) | 51.7 | (25.4) | 3.0 | Student's t-test | ns | ns |
| Chan et al. 2010 [29] | Baseline diabetes mellitus | Report | KCCQ SS 6-month change |  |  |  |  |  | Bivariate linear regression model ^(i)^ | ns | ns |
| Comín-Colet at al. 2016 [30] | Diabetes mellitus | Report | KCCQ SS <50 (worse) |  |  |  |  |  | Univariable binary logistic regression | ≤.05 | Worse HRQoL |
|  |  |  | KCCQ SS |  |  |  |  |  | Univariable linear regression | ≤.001 | Worse HRQoL |
|  |  |  | KCCQ SS |  |  |  |  |  | Multivariable linear regression ^(ii)^ | ns | ns |
|  |  |  | EQ-5D Index <0.5 (worse) |  |  |  |  |  | Univariable binary logistic regression | ≤.001 | Worse HRQoL |
|  |  |  | EQ-5D Index |  |  |  |  |  | Univariable linear regression | ≤.001 | Worse HRQoL |
|  |  |  | EQ-5D Index |  |  |  |  |  | Multivariable linear regression ^(ii)^ | ns | ns |
|  |  |  | EQ-5D VASS <50 (worse) |  |  |  |  |  | Univariable binary logistic regression | ≤.05 | Worse HRQoL |
|  |  |  | EQ-5D VASS |  |  |  |  |  | Univariable linear regression | ≤.001 | Worse HRQoL |
|  |  |  | EQ-5D VASS |  |  |  |  |  | Multivariable linear regression ^(ii)^ | ns | ns |
| Comín-Colet et al. 2013 [14] | Diabetes mellitus | Report | MLHFQ SS ≥ median [42] (worse) |  |  |  |  |  | Univariable binary logistic regression | ≤.001 | Worse HRQoL |
|  |  |  | MLHFQ SS |  |  |  |  |  | Multivariate regression ^(iii)^ | ≤.001 | Worse HRQoL |
|  |  |  | MLHFQ PS |  |  |  |  |  | Multivariate regression ^(iii)^ | ≤.05 | Worse HRQoL |
|  |  |  | MLHFQ ES |  |  |  |  |  | Multivariate regression ^(iii)^ | ≤.001 | Worse HRQoL |
| Enjuanes et al. 2014 [32] | Diabetes mellitus | Report | MLHFQ SS |  |  |  |  |  | Univariate linear regression | ≤.001 | Worse HRQoL |
|  |  |  | MLHFQ SS |  |  |  |  |  | Multivariable linear regression ^(iv)^ | ≤.01 | Worse HRQoL |
| Fotos et al. 2013 [16] | Diabetes mellitus | Report | MLHFQ SS | 56.9 | (19.8) | 69.3 | (19.0) | 21.8 | Student's t-test | ≤.001 | Worse HRQoL |
|  |  |  | MLHFQ SS |  |  |  |  |  | Multivariate linear regression ^(v)^ | ≤.001 | Worse HRQoL |
| Fritschi & Redeker 2015 [33] | Diabetes mellitus | Report | SF-36 SS | 13.5 | (0.4) | 11.8 | (0.6) | -12.4 | Univariate general linear model ^(vi)^ | ≤.05 | Worse HRQoL |
|  |  |  | SF-36 PS | 26.6 | (0.1) | 26.1 | (0.2) | -1.9 | Univariate general linear model ^(vi)^ | ≤.05 | Worse HRQoL |
| Harrow et al. 2011 [35] | Diabetes mellitus + medication | Report | SF-6D HU 36m change |  |  |  |  |  | Multiple linear regression ^(vii)^ | ns | ns |
| Iqbal et al. 2010 [36] | Diabetes mellitus (%) ^(a)^ | Report | Good/poor HRQoL ^(B)^ |  |  |  |  |  | Chi-square/Fisher's exact test | ns | ns |
|  |  |  | MLHFQ SS |  |  |  |  |  | Multiple regression ^(viii)^ | ns | ns |
| Smolderen et al. 2009 [38] | Diabetes mellitus | Report | SF-12 PS |  |  |  |  |  | Multivariable linear regression ^(ix)^ | ≤.001 | Worse HRQoL |
|  |  |  | SF-12 MS |  |  |  |  |  | Multivariable linear regression ^(ix)^ | ns | ns |
| Streng et al. 2018 [8] | Diabetes mellitus | Report | KCCQ SS | 47.0 | [31–64] | 40.0 | [26–27] | -14.9 | Univariable linear regression | ≤.001 | Worse HRQoL |
|  |  |  | KCCQ SS |  |  |  |  |  | Multivariable linear regression ^(x)^ | ≤.001 | Worse HRQoL |
|  |  |  | EQ-5D VASS | 60.0 | [45–70] | 50.0 | [40–70] | -16.7 | Univariable linear regression | ≤.001 | Worse HRQoL |

ES (emotional score), HRQoL (health-related quality of life) HU (health utilities), KCCQ (Kansas City Cardiomyopathy Questionnaire), MLHFQ (Minnesota Living with Heart Failure Questionnaire), MS (mental score), PS (physical score), SS (summary score), VASS (visual analogue scale score). Missing information accounts for a lack of data availability within selected studies.

**^(a)^** Comorbidity prevalence as a function of outcome

**^(A)^** Poor outcome (death or average KCCQ summary score <45 throughout one-year post-LVAD) or acceptable outcome (one-year survival with KCCQ ≥45); ^(B)^ MLHFQ SS <median (good) or ≥median (poor)

**^(i)^** Adjusted for baseline KCCQ; **^(ii)^** including age, sex, body mass index (BMI), systolic blood pressure (SBP), heart rate, New York Heart Association (NYHA) class, left ventricular ejection fraction (LVEF), Charlson comorbidity Index, ischemic etiology, estimated glomerular filtration rate (eGFR), hypertension, atrial fibrillation, diabetes mellitus, Hb, optimal treatment, inclusion service, recent admission, and time since diagnosis; **^(iii)^** including anemia and iron deficiency, and adjusted for age, gender, LVEF, SBP, heart rate ≥70, CKD, hospital admission related to HF in the previous 6 months, ischemic chronic HF etiology, dependency [Barthel Index <90], hypertension, diabetes mellitus, angiotensin converting enzyme inhibitors (ACEI) or angiotensin II receptor blockers (ARB), loop diuretics, N-terminal pro b-type natriuretic peptide (NT-proBNP) ≥median [1620pg/mL], c-reactive protein (CRP) ≥median [0.8mg/dL], NYHA class [II-III and IV], anemia [Hb≤12g/dL], iron deficiency, and sTfR ≥median [1.62mg/L]; **^(iv)^** including iron deficiency and anemia adjusted for age, gender, LVEF, center [Poland vs Holland and Spain vs Holland], BMI <18.5kg/m2, SBP, heart rate, NYHA class [II-III vs I and IV vs I], preserved [<45%] vs reduced LVEF, LVEDD >55mm, HF etiology [ischemic vs non ischemic], hypertension, diabetes mellitus, anemia, iron deficiency, ACEi or ARB, aldosterone antagonists, statins, loop diuretics, antiplatelets, anticoagulants, Hb, transferrin saturation (TSAT), red cell distribution width (RDW), mean corpuscular hemoglobin (MCH), RDW>15% + MCV≤100fl, NT-proBNP, eGFR, and CRP; **^(v)^** including age, marital status, NYHA class [III or IV], diabetes mellitus, hypertension, chronic renal failure, chronic respiratory failure, cancer, psychiatric disease, cardiac interventions in the last 6 months, hospitalizations in the last year, and taking medication at the right time; **^(vi)^** adjusted for age, gender, NYHA class, BMI; **^(vii)^** including Hb change group (loss vs no loss), age, race, education level, BMI category, diabetes with medications, disability, rheumatoid arthritis without medications, hospitalized in the past 2 years, exercise level (METS), depression score, stroke or heart attack, cancer diagnosis, nonsteroidal anti-inflammatory drug use, and baseline Hb group; **^(viii)^** including age, male, SIMD, having a carer, NYHA (I-II and III-IV), chronic obstructive pulmonary disease, diabetes, beta-blockers, spironolactone, carer male, carer Scottish Index of Multiple Deprivation score, carer EQ-5D score, carer full time job; **^(ix)^** including age, gender, current smoking, hypercholesterolemia, hypertension, diabetes mellitus; adjusted for cardiac history, LVEF, history of peripheral artery disease, having no partner, and low education; **^(x)^** adjusted for age, sex, and NYHA class

**^(I)^** Chronic heart failure patients with poor outcomes have greater prevalence of diabetes mellitus

**Table 10.** Respiratory diseases and quality of life

|  | **Comorbidity** | **Definition** | **Quality of life assessment** | **Without comorbidity** | | **With**  **comorbidity** | | **Δ**  **(%)** | **Statistical test** | ***P*** | **Interpretation** |
| --- | --- | --- | --- | --- | --- | --- | --- | --- | --- | --- | --- |
| Arnold et al. 2016 [26] | Baseline lung disease (%) ^(a)^ | Report | Poor/acceptable outcome^(A)^ |  |  |  |  |  | Chi-square test | ns | ns |
| Bektas et al., 2017 [18] | COPD | FEV1/FVC <0.70 after bronchodilatation | KCCQ SS | 82.0 | (19.0) | 73.0 | (21.0) | -11.0 | Student's t-test | ≤.01 | Worse HRQoL |
|  | COPD | FEV1/FVC <0.70 after bronchodilatation | KCCQ PLS | 54.0 | (15.0) | 47.0 | (16.0) | -13.0 | Student's t-test | ≤.01 | Worse HRQoL |
|  | COPD | FEV1/FVC <0.70 after bronchodilatation | KCCQ SyS | 79.0 | (24.0) | 67.0 | (22.0) | -15.2 | Student's t-test | ≤.001 | Worse HRQoL |
|  | COPD | FEV1/FVC <0.70 after bronchodilatation | KCCQ SySS | 46.0 | (13.0) | 41.0 | (13.0) | -10.9 | Student's t-test | ≤.05 | Worse HRQoL |
|  | COPD | FEV1/FVC <0.70 after bronchodilatation | KCCQ SLS | 64.0 | (21.0) | 58.0 | (20.0) | -9.4 | Student's t-test | ns | ns |
|  | COPD | FEV1/FVC <0.70 after bronchodilatation | KCCQ SES | 80.0 | (17.0) | 74.0 | (21.0) | -7.5 | Student's t-test | ns | ns |
|  | COPD | FEV1/FVC <0.70 after bronchodilatation | KCCQ QoLS | 67.0 | (19.0) | 61.0 | (19.0) | -9.0 | Student's t-test | ns | ns |
| Carson et al. 2009 [28] | COPD | Report | MLHFQ SS | 49.9 | 25 | 54.8 | 25.8 | 9.8 | Student's t-test | ≤.05 | Worse HRQoL |
|  |  |  | MLHFQ SS |  |  |  |  |  | Multivariable regression **^(i)^** | ≤.001 | Worse HRQoL |
| Fotos et al. 2013 [16] | Chronic respiratory failure | Report | MLHFQ SS | 59.5 | 19.5 | 72.2 | 20 | 21.3 | Student's t-test | ≤.001 | Worse HRQoL |
|  |  |  | MLHFQ SS |  |  |  |  |  | Multivariate linear regression **^(ii)^** | ≤.001 | Worse HRQoL |
| Iqbal et al. 2010 [36] | COPD (%) ^(a)^ | Report | Good/poor HRQoL^(B)^ |  |  |  |  |  | Chi-square or Fisher's exact test | ns | ns |
|  |  |  | MLHFQ SS |  |  |  |  |  | Multiple regression **^(iii)^** | ns | ns |
| Streng et al. 2018 [8] | COPD | Report | KCCQ SS | 46.0 | [31–63] | 36.0 | [24–49] | -21.7 | Univariable linear regression | ≤.001 | Worse HRQoL |
|  |  |  | KCCQ SS |  |  |  |  |  | Multivariable linear regression **^(iv)^** | ≤.001 | Worse HRQoL |
|  | COPD | Report | EQ-5D VASS | 60.0 | [45–70] | 50.0 | [40–65] | -16.7 | Univariable linear regression | ≤.001 | Worse HRQoL |

COPD (chronic obstructive pulmonary disease), FEV1 (forced expiratory volume in one second), FVC (forced vital capacity), HRQoL (health-related quality of life), KCCQ (Kansas City Cardiomyopathy Questionnaire), MLHFQ (Minnesota Living with Heart Failure Questionnaire), PLS (physical limitation score), QoLS (quality of life score), SES (self-efficacy score), SLS (social limitation score), SS (summary score), SyS (symptom score), SySS (symptom stability score), VASS (visual analogue scale score). Missing information accounts for a lack of data availability within selected studies.

**^(a)^** Comorbidity prevalence as a function of outcome

**^(A)^** Poor outcome (death or average KCCQ summary score <45 throughout one-year post-LVAD) or acceptable outcome (one-year survival with KCCQ ≥45); **^(B)^** MLHFQ SS <median (good) or ≥median (poor)

**^(i)^** adjusted for baseline SBP and DBP, heart rate, age, weight, ejection fraction, LVIDD, ischemic, idiopathic, hypertensive and valvular etiologies, diabetes mellitus, atrial fibrillation, arrythmias, ventricular tachycardia, hyperlipidemia, COPD, previous myocardial infarction, history of hypertension, LnBNP (log-transformed B-type natriuretic peptide), creatinine, use of beta-blockers, angiotensin converting enzyme inhibitors, angiotensin II receptor blockers, aldosterone antagonists, diuretics, and digitalis glycosides; **^(ii)^** including age, marital status, New York Heart Association (NYHA) class [III or IV], diabetes mellitus, hypertension, chronic renal failure, chronic respiratory failure, cancer, psychiatric disease, cardiac interventions in the last 6 months, hospitalizations in the last year, and taking medication at the right time; **^(iii)^** including age, male, Scottish Index of Multiple Deprivation (SIMD), having a carer, NYHA (I-II and III-IV), COPD, diabetes, beta-blockers, spironolactone, carer male, carer SIMD score, carer EQ-5D score, carer full time job; **^(iv)^** adjusted for age, sex, and NYHA class
