# Supplementary material for: Impact of non-cardiovascular comorbidities on the quality of life of patients with chronic heart failure: a scoping review
Source: Health Qual Life Outcomes. 2020 Oct 7;18:329. doi: 10.1186/s12955-020-01566-y (PMC7542693; doi:10.1186/s12955-020-01566-y)
Supplement: Supplementary file 3 — Additional file 3: Risk of bias for individual studies. Risk of bias grading for individual studies according to the Quality Assessment Tool for Observational Cohort and Cross-Sectional Studies [file 12955_2020_1566_MOESM3_ESM.docx]

**Additional file 3** Risk of bias for individual studies according to the Quality Assessment Tool for Observational Cohort and Cross-Sectional Studies [25]

| **Reference** | **Q1** | **Q2** | **Q3** | **Q4** | **Q5** | **Q6** | **Q7** | **Q8** | **Q9** | **Q10** | **Q11** | **Q12** | **Q13** | **Q14** | **Rating** |
| --- | --- | --- | --- | --- | --- | --- | --- | --- | --- | --- | --- | --- | --- | --- | --- |
| Ancheta et al. 2009 [17] | No | No | NR/CD | NR/CD | No | No | No | Yes | Yes | NA | Yes | No | NA | Yes/No | Fair |
| Arnold et al. 2016 [26] | Yes | Yes | Yes | Yes | No | Yes | Yes | No | NR/CD | NA | Yes | No | Yes | No/NR | Good |
| Bektas et al. 2017 [18] | No | Yes | NR/CD | Yes | No | No | No | Yes | Yes | NA | No | NR/CD | NA | No/NR | Fair |
| Bhatt et al. 2016 [27] | Yes | Yes | Yes | Yes | No | No | No | Yes | Yes | NA | Yes | No | NA | Yes | Good |
| Carson et al. 2009 [28] | No | No | NR/CD | NR/CD | No | No | No | No | No/CD | NA | Yes | No | NA | Yes/No | Fair |
| Chan et al. 2010 [29] | Yes | Yes | Yes | Yes | No | Yes | Yes | No | No | NA | Yes | NR/CD | Yes | Yes | Good |
| Comín-Colet et al. 2016 [30] | Yes | Yes | NR/CD | Yes | No | No | No | Yes/No | No/CD | NA | Yes | No | NA | Yes/No | Fair |
| Comín-Colet et al. 2013 [14] | Yes | No | Yes | NR/CD | No | No | No | Yes/No | Yes/No | NA | Yes | No/NR/CD | NA | Yes/No | Fair |
| Cully et al. 2010 [31] | Yes | No | Yes | NR/CD | No | No | No | Yes | Yes | NA | No | No | NA | No/NR | Fair |
| Enjuanes et al. 2014 [32] | Yes | No | NR/CD | No | No | No | No | Yes/No | Yes/No | NA | Yes | No/NR/CD | NA | Yes | Fair |
| Fotos et al. 2013 [16] | Yes | Yes | Yes | Yes | No | No | No | No | No/CD | NA | Yes | No | NA | Yes | Fair |
| Fritschi & Redeker 2015 [33] | Yes | No | Yes | NR/CD | No | No | No | No | No | NA | No | No | NA | Yes | Fair |
| Gastelurrutia et al. 2013 [34] | Yes | Yes | NR/CD | Yes | No | No | No | Yes | No | NA | Yes | No | NA | Yes | Fair |
| Harrow et al. 2011 [35] | Yes | Yes | No | Yes | No | Yes | Yes | Yes/No | NR/CD | NA | Yes | NR/CD | No | Yes | Good |
| Iqbal et al. 2010 [36] | Yes | No | NR/CD | NR/CD | No | No | No | No | NR/CD | NA | Yes | No | NA | Yes/No | Fair |
| Moliner et al. 2017 [37] | Yes | No | NR/CD | No | No | No | No | Yes/No | Yes/No | NA | Yes | No/NR/CD | NA | Yes | Fair |
| Pantilant et al. 2016 [15] | Yes | Yes | Yes | Yes | No | No | No | Yes | Yes | NA | Yes | No | NA | Yes | Good |
| Smolderen et al. 2009 [38] | Yes | Yes | Yes | Yes | No | No | No | No | No/CD | NA | Yes | No | NA | Yes | Fair |
| Staniute et al. 2015 [39] | Yes | Yes | NR/CD | Yes | No | No | No | Yes | Yes | NA | Yes | No | NA | Yes | Good |
| Streng et al. 2018 [8] | Yes | No | Yes | No | No | No | No | Yes/No | NR/CD | NA | No | No/NR/CD | NA | Yes/No | Fair |
| Weinbergen et al. 2018 [40] | Yes | Yes | NR/CD | Yes | No | Yes | Yes | Yes | Yes | NA | Yes | NR/CD | Yes | No/NR | Good |

Q (question), NR (not reported), CD (cannot determine), NA (not applicable) **Q1.** Was the research question or objective in this paper clearly stated? **Q2.** Was the study population clearly specified and defined? **Q3.** Was the participation rate of eligible persons at least 50%? **Q4.** Were all the subjects selected or recruited from the same or similar populations (including the same time period)? Were inclusion and exclusion criteria for being in the study prespecified and applied uniformly to all participants? **Q5.** Was a sample size justification, power description, or variance and effect estimates provided? **Q6.** For the analyses in this paper, were the exposure(s) of interest measured prior to the outcome(s) being measured? **Q7.** Was the timeframe sufficient so that one could reasonably expect to see an association between exposure and outcome if it existed? **Q8.** For exposures that can vary in amount or level, did the study examine different levels of the exposure as related to the outcome (e.g., categories of exposure, or exposure measured as continuous variable)? **Q9.** Were the exposure measures (independent variables) clearly defined, valid, reliable, and implemented consistently across all study participants? **Q10.** Was the exposure(s) assessed more than once over time? **Q11.** Were the outcome measures (dependent variables) clearly defined, valid, reliable, and implemented consistently across all study participants? **Q12.** Were the outcome assessors blinded to the exposure status of participants? **Q13.** Was loss to follow-up after baseline 20% or less? **Q14.** Were key potential confounding variables measured and adjusted statistically for their impact on the relationship between exposure(s) and outcome(s)? [25]
